# Ezh2 programs T_FH_ differentiation by integrating phosphorylation-dependent activation of Bcl6 and polycomb-dependent repression of p19Arf

**DOI:** 10.1038/s41467-018-07853-z

**Published:** 2018-12-21

**Authors:** Fengyin Li, Zhouhao Zeng, Shaojun Xing, Jodi A. Gullicksrud, Qiang Shan, Jinyong Choi, Vladimir P. Badovinac, Shane Crotty, Weiqun Peng, Hai-Hui Xue

**Affiliations:** 1 Institutes for Translational Medicine, Soochow University, Suzhou, Jiangsu P. R. China 215123; 2 Department of Microbiology and Immunology, Carver College of Medicine, University of Iowa, Iowa City, IA USA 52242; 3 Department of Physics, The George Washington University, Washington DC, 20052 USA; 4 Department of Pathogen Biology, Shenzhen University School of Medicine, Shenzhen, Guangdong P. R. China 518071; 5 La Jolla Institute, La Jolla, CA USA 92037; 6 Department of Pathology, Carver College of Medicine, University of Iowa, Iowa City, IA USA 52242; 7 Present Address: Department of Pathobiology, School of Veterinary Medicine, University of Pennsylvania, Philadelphia, PA 19104 USA

## Abstract

Ezh2 is an histone methyltransferase (HMT) that catalyzes H3K27me3 and functions in T_H_1, T_H_2, and Treg cells primarily via HMT activity. Here we show that Ezh2 ablation impairs T follicular helper (T_FH_) cell differentiation and activation of the T_FH_ transcription program. In T_FH_ cells, most Ezh2-occupied genomic sites, including the *Bcl6* promoter, are associated with H3K27ac rather than H3K27me3. Mechanistically, Ezh2 is recruited by Tcf1 to directly activate *Bcl6* transcription, with this function requiring Ezh2 phosphorylation at Ser21. Meanwhile, Ezh2 deploys H3K27me3 to repress *Cdkn2a* expression in T_FH_ cells, where aberrantly upregulated p19Arf, a *Cdkn2a* protein product, triggers T_FH_ cell apoptosis and antagonizes Bcl6 function via protein-protein interaction. Either forced expression of Bcl6 or genetic ablation of p19Arf in Ezh2-deficient cells improves T_FH_ cell differentiation and helper function. Thus, Ezh2 orchestrates T_FH_-lineage specification and function maturation by integrating phosphorylation-dependent transcriptional activation and HMT-dependent gene repression.

## Abstract (web summary)

Ezh2 is an histone methyltransferase that catalyzes H3K27me3. Here the authors show that Ezh2 promotes T follicular helper (T_FH_) differentiation and helper activity, by cooperating with Tcf1 to activate Bcl6 transcription and epigenetically repressing p19Arf, an antagonist of Bcl6 function and T_FH_ cell survival.

## Introduction

T follicular helper (T_FH_) cells are specialized CD4^+^ T cells that provide essential help to humoral immunity. In physiological responses to infections, T_FH_ cells provide costimulatory molecules and cytokines, prompting B cells to undergo somatic hypermutation and affinity maturation, and transition into plasma cells and memory B cells^1,2^. T_FH_ differentiation is a multi-stage process that involves a number of transcription factors (TFs) that drive T_FH_ lineage specification and functional maturation^3,4^. Bcl6 is the T_FH_ lineage-defining TF induced in all T_FH_ cells during an early lineage-specification stage, and maintained at elevated levels as the cells mature to germinal center (GC) T_FH_ cells^5,6^. Other TFs, including Tcf1 and Lef1^7–9^, Stats^10,11^, Maf^12,13^, Batf^14^, Irf4^15^, and Acsl2^16^, also promote T_FH_ differentiation, whereas Foxo1^17^, Klf2^18,19^, and Foxp1^20^ factors negatively regulate T_FH_ responses. The extent to which epigenetic mechanisms might control the transcriptional events that drive the T_FH_ differentiation program is unknown.

TFs use epigenetic mechanisms to establish cell identity and maintain heritable gene expression patterns^21,22^. The epigenetic regulator polycomb repressive complex 2 (PRC2) is comprised of multiple subunits, including Ezh2, Suz12, and Eed^23,24^, with Ezh2 providing the histone methyltransferase (HMT) activity that catalyzes histone H3 trimethylation at lysine 27 (H3K27me3). H3K27me3 is a repressive histone mark, associated with chromatin compaction and gene silencing. In CD4^+^ T cells, Ezh2 critically regulates cytokine production and plasticity of in vitro polarized T helper 1 (T_H_1) and T_H_2 cells^25–29^, sustains T cell responses in vivo^30,31^, and maintains regulatory T (Treg) cell identity and repressive capacity^32–34^. Nevertheless, whether and how Ezh2 contributes to regulation of T_FH_ differentiation is unknown.

In T cells, Ezh2 function is largely attributed to HMT-mediated gene repression. For example, Ezh2 represses T_H_2 lineage-defining Gata3 TF in T_H_1 cells, but instead represses T_H_1 lineage-defining T-bet TF in T_H_2 cells, by deploying H3K27me3 to these loci^28^. Yet, some genes bound by Ezh2 lack H3K27me3, as shown in high throughput studies of T_H_1, T_H_2 and Treg cells, and their expression are downregulated, rather than upregulated, upon loss of Ezh2^28,32,33^. The underlying mechanisms are unknown but likely include indirect effects, such as upregulation of repressive factor(s), or Ezh2-mediated gene activation, as observed in malignantly transformed cells^35,36^. More importantly, it is not clear whether Ezh2 activates gene expression to regulate T cells in vivo.

In this study, we address the knowledge gaps noted above by conditionally targeting Ezh2 in T cells, coupled with analyses of T_FH_ differentiation elicited by viral infection and protein immunization. Our data indicate that Ezh2 is rapidly induced and phosphorylated at Ser21 in activated CD4^+^ T cells. Following T_H_1 and T_FH_ lineage bifurcation, Ser21-phosphorylated Ezh2 is predominantly associated with T_FH_ cells, where it activates transcription of Bcl6 and a broad T_FH_ transcription program. On the other hand, the Ezh2 HMT activity, regardless of Ser21 phosphorylation status, critically represses *Cdkn2a* products to promote T_FH_ cell survival and differentiation. These findings identify Ezh2 as an integrator of epigenetic and transcriptional regulatory mechanisms to program T_FH_ fate decision, survival, and functional maturation.

## Results

### Ezh2 is essential for T_FH_ responses to viral infection

**Fig. 1:**
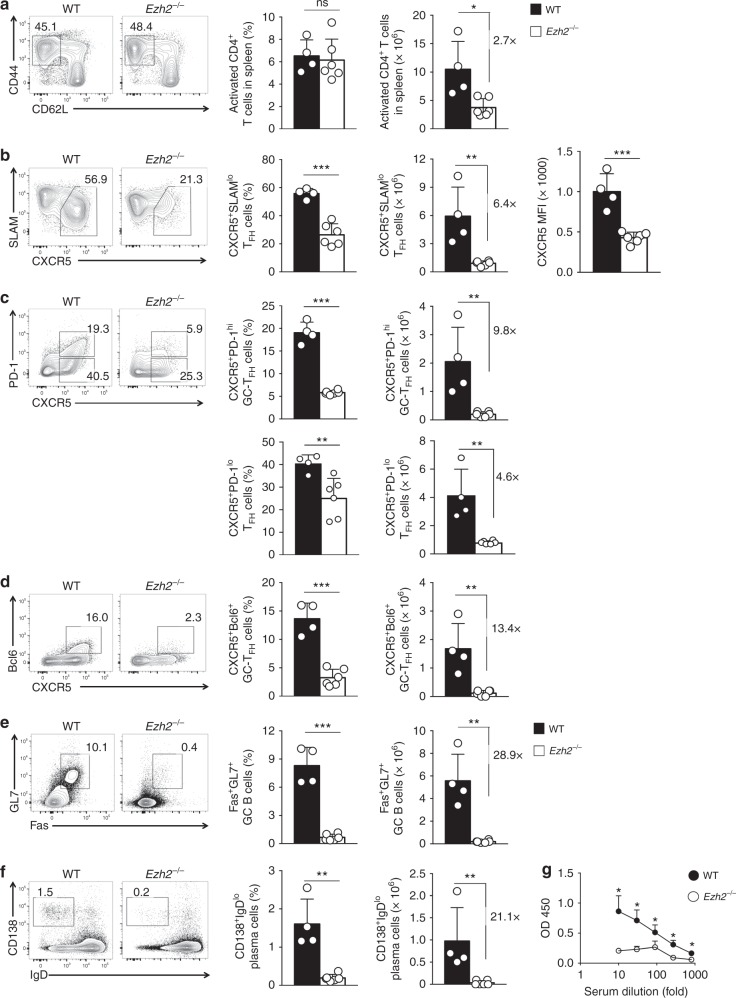
Ezh2 is required for T_FH_ and B cell responses to viral infection. **a**–**f** T cell-specific ablation of Ezh2 affects T_FH_ and B cell responses to viral infection. WT and *Ezh2*^–/–^ mice were infected with VacV. On 8 *dpi*, CD44^hi^CD62L^–^ activated CD4^+^ T cells were detected in the spleen (**a**), and further analyzed for CXCR5^+^SLAM^lo^ T_FH_ cells and CXCR5 expression levels (**b**), CXCR5^+^PD-1^lo^ T_FH_ and CXCR5^+^PD-1^hi^ GC-T_FH_ cells (**c**), and CXCR5^+^Bcl6^+^ T_FH_ cells (**d**). Splenic B220^+^CD19^+^ cells were analyzed for Fas^+^GL7^+^ GC B cells (**e**) and CD138^+^IgD^lo^ plasma cells (**f**). Contour plots are representative of two experiments, and cumulative data on frequency and numbers of each subset are the means ± s.d., and each dot represents a mouse. (**g**) Detection of VacV-specific IgG in sera of infected mice collected on 8 dpi (*n* = 4 for WT and 5 for *Ezh2*^–/–^). *p < 0.05; **p < 0.01; ***p < 0.001 by unpaired two-tailed Student’s *t*-test

To investigate the requirements for Ezh2 in T_FH_ cells, we used *CD4-Cre* to ablate *Ezh2* in T cells. Consistent with previous studies^29,30^, *CD4-Cre*^+^*Ezh2*^fl/fl^ (*Ezh2*^–/–^) mice showed no detectable abnormalities in T cell homeostasis. We then infected *Ezh2*^–/–^ mice or littermate controls with vaccinia virus (VacV). On day 8 post-infection (8 *dpi*), both wild-type and *Ezh2*^–/–^ CD4^+^ T cells were activated at similar frequency, but fewer activated CD4^+^ T cells were detected in *Ezh2*^–/–^ mice (Fig. 1a). In *Ezh2*^–/–^ mice, among the CD44^hi^CD62L^–^ activated CD4^+^ T cells, CXCR5^+^SLAM^lo^ T_FH_ cells were more profoundly reduced (Fig. 1b), and CXCR5 expression was also diminished (Fig. 1b). In addition, generation of CXCR5^+^PD-1^lo^ T_FH_ cells was impaired, and production of CXCR5^+^Bcl6^+^ and CXCR5^+^PD-1^hi^ GC T_FH_ (GC-T_FH_) cells was almost completely abrogated (Fig. 1c, d). Similar defects in T_FH_ differentiation were observed in the *Ezh2*^–/–^ mice infected by the Armstrong strain of lymphocytic choriomeningitis virus (LCMV-Arm, Supplementary Figure 1a–d). These observations demonstrate a conserved requirement for Ezh2 in T_FH_ cell responses to acute viral infections. In addition, *Ezh2*^–/–^ mice generated few Fas^+^GL7^+^ GC B cells and CD138^+^IgD^lo^ plasma cells in response to infections by VacV and LCMV (Fig. 1e, f, Supplementary Figure 1e, f), and produced little VacV-specific antibody (Fig. 1g). These data further corroborate an essential role of Ezh2 in generating functionally competent T_FH_ cells.

### Ezh2 is critical for activation of T_FH_ transcription program

**Fig. 2:**
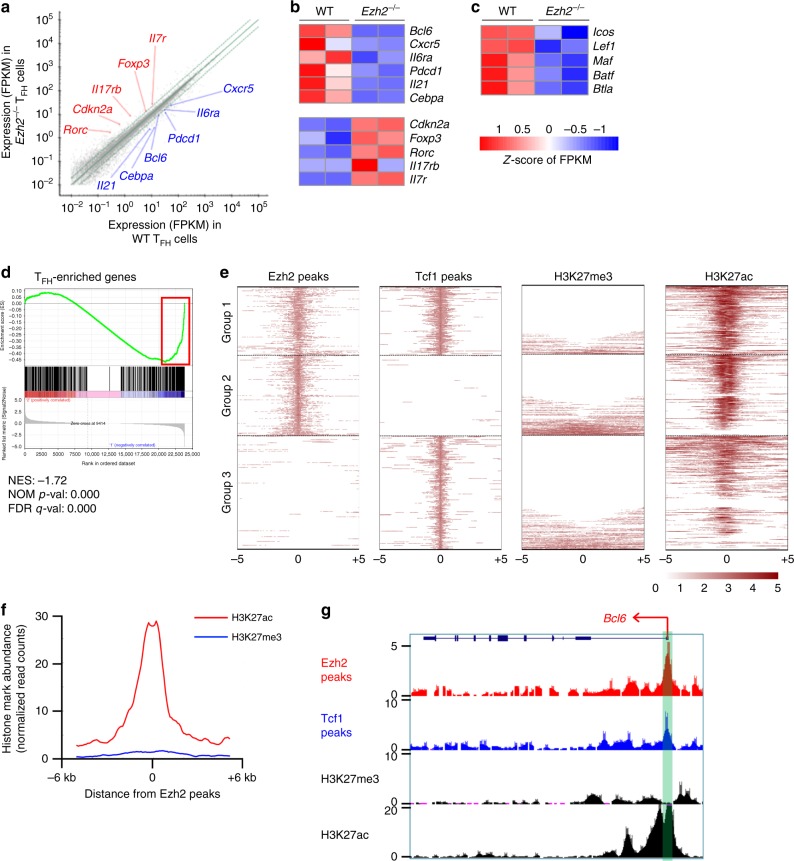
Ezh2 activates the T_FH_ transcriptional program. **a** RNA-Seq analysis of *Ezh2*^–/–^ or WT CXCR5^+^PD-1^lo^ T_FH_ cells sort-purified on day 8 after VacV infection. Scatterplot shows the average FPKM values of two replicates of WT vs. *Ezh2*^–/–^ T_FH_ cells, with green lines denoting gene expression changes by a factor of 2. Select differentially expressed genes are marked, with genes upregulated in *Ezh2*^–/–^ T_FH_ cells in red and those downregulated ones in blue. **b**–**c** Heatmaps of select differentially expressed genes with ≥2 fold (**b**), or <2 fold but ≥1.5-fold expression changes (**c**), all with an FDR < 0.05. (**d**) GSEA enrichment plot shows T_FH_-associated gene set (from Choi et al.^7^) is negatively enriched in *Ezh2*^–/–^ T_FH_ cells. NES, normalized enrichment scores, NOM *p*-val, nominal *p* values, FDR q-val, false discovery rate *q* values. Heatmap of the negatively enriched genes at the leading edge (highlighted in red rectangle) is shown in Supplementary Figure 2a. **e** Heatmaps of Ezh2, Tcf1, H3K27me3, and H3K27ac ChIP-Seq signals, at +/–5 kb around the Ezh2 peak summit (for Ezh2 and Tcf1 co-occupied sites in group1, and for Ezh2 solo sites in group 2) or Tcf1 peak summit (for the Tcf1 solo sites in group 3) in WT T_FH_ cells. WT C57BL/6 mice were infected with VacV, and on 8 dpi, CXCR5^+^PD-1^lo^ T_FH_ cells were sorted from the spleens and analyzed by ChIP-Seq. In each group, the Ezh2 and Tcf1 common or solo peaks were clustered according to H3K27me3 signals (from low to high), and the H3K27me3^–^ peaks were further clustered according to H3K27ac signals (from high to low). The color scale indicates average signals using a 100-base pair window. **f** The profile of H3K27me3 and H3K27ac at 51 Ezh2 peaks found within +/–10 kb regions flanking the TSSs of 47 genes identified at the leading edge of GSEA enrichment plot in (**d**). **g** ChIP-Seq tracks of Ezh2, Tcf1, H3K27me3, and H3K27ac at the *Bcl6* gene locus as displayed on the UCSC Genome Browser, with the vertical green bar denoting the Ezh2/Tcf1 co-occupied site

We next investigated how Ezh2 deficiency affected the T_FH_ transcriptome. To avoid changes in cell composition, CXCR5^+^PD-1^lo^ T_FH_ cells were specifically sorted from VacV-infected WT and *Ezh2*^–/–^ mice on 8 dpi and analyzed by RNA-Seq. By setting a cut-off at ≥2-fold expression changes and FDR < 0.05, 40 upregulated and 105 downregulated genes were identified in *Ezh2*^–/–^ T_FH_ cells. The upregulated genes included *Cdkn2a*, *Foxp3* and *Rorc* (Fig. 2a, b). The downregulated genes included *Bcl6*, the T_FH_-characteristic cytokine *Il21*, and the surface receptors, *Cxcr5* and *Pdcd1* (Fig. 2a, b). In the *Ezh2*^–/–^ T_FH_ cells, expression of key T_FH_ regulators (i.e., *Maf, Lef1*, and *Icos*) decreased by ≥1.5-fold but less than two-fold (Fig. 2c). Previously, we defined a T_FH_-enriched gene set that contains 491 genes^7^. By gene set enrichment analysis (GSEA), which does not set a threshold for expression changes but assesses the behavior of a given set of functionally related genes, the T_FH_-enriched gene set were negatively enriched in *Ezh2*^–/–^ T_FH_ cells, with 130 genes at the leading edge of the enrichment plot and showing diminished expression (Fig. 2d, Supplementary Figure 2a). These analyses suggest that loss of Ezh2 leads to compromised activation of a broad T_FH_ transcription program.

To strengthen the connection between Ezh2 and induction of the T_FH_ program, we tested whether Ezh2 could activate these T_FH_ genes, directly. To this end, ChIP-Seq of Ezh2, H3K27me3, and H3K27ac was performed on sorted WT CXCR5^+^PD-1^lo^ T_FH_ cells elicited by VacV infection. Consistent with existing knowledge^37^, the signal strength of H3K27me3 was negatively correlated, whereas that of H3K27ac was positively correlated with gene expression levels in T_FH_ cells (Supplementary Figure 2b, c). Using the SICER algorithm^38^ at a setting of FDR < 10^–4^, 6,130 Ezh2 peaks were identified, but surprisingly, only a small portion were associated with H3K27me3. In contrast, most Ezh2 peaks were correlated with H3K27ac (Fig. 2e). Among the 130, Ezh2-dependent, T_FH_-enriched genes (defined by GSEA in Fig. 2d), 47 harbored one or more Ezh2 peaks, within 10 kb of the transcription start sites (TSSs), and these Ezh2 peaks associated strongly with H3K27ac but little with H3K27me3 (Fig. 2f). Ezh2 peaks were located at the promoter regions (defined as +/−1 kb regions flanking the TSSs) of *Bcl6*, *Icos*, *Maf* and *Il21*, an upstream region of *Cxcr5*, and an intronic region of *Icos* (Fig. 2g, Supplementary Figure 2d). All of these Ezh2-occupied sites and corresponding gene loci exhibited strong H3K27ac signals, with H3K27me3 detected at no more than background levels (Fig. 2g, Supplementary Figure 2d). These results strongly suggested that, in T_FH_ cells, the predominant role of Ezh2 is to directly activate gene expression, with Ezh2-conferred H3K27me3 modifications occurring at fewer gene loci.

### Ezh2 acts upstream of Bcl6 to promote T_FH_ differentiation

We next investigated functional requirements for Ezh2-mediated induction of Bcl6. To control for potential alterations in precursor frequency and facilitate molecular characterization of Ezh2-deficient T_FH_ cells, *Ezh2*^–/–^ mice were crossed with a Smarta TCR transgenic strain that expresses an MHC II-restricted TCR specifically recognizing the LCMV GP61 epitope. WT or *Ezh2*^–/–^ Smarta CD4^+^ T cells were adoptively transferred into congenic recipients, followed by LCMV-Arm infection. *Ezh2*^–/–^ Smarta CD4^+^ T cells showed reduced expansion from 4 dpi, and the defect became more profound on 6 and 8 dpi, partly due to apoptosis (Supplementary Figure 3a, b).

**Fig. 3:**
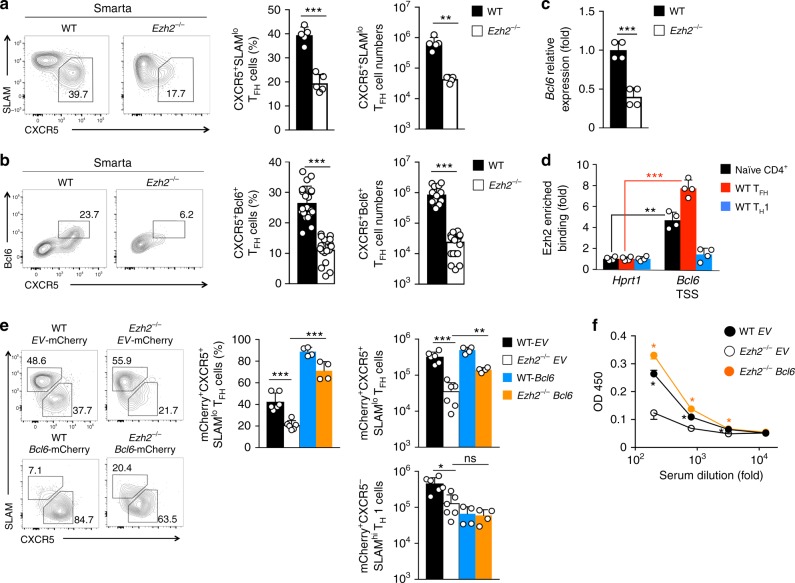
Ezh2 acts upstream of Bcl6 induction to promote T_FH_ differentiation. **a**–**b** Impact of Ezh2 deficiency on monoclonal CD4^+^ T cell responses. CD45.2^+^ Smarta CD4^+^ T cells from WT or *Ezh2*^–/–^ Smarta-Tg mice were adoptively transferred into congenic mice, followed by infection with LCMV-Arm. On 4 dpi, CXCR5^+^SLAM^lo^ (**a**) and CXCR5^+^Bcl6^+^ T_FH_ cells (**b**) were detected in recipient spleens. Contour plots are representative of ≥2 experiments, and cumulative data on frequency and numbers of each subset are means ± s.d. (each dot represents a mouse). **c** Detection of *Bcl6* transcripts in T_FH_ cells. CD45.2^+^ CXCR5^+^ SLAM^lo^ T_FH_ cells were sorted from the recipient spleens on 4 dpi as in (**a**), and *Bcl6* transcript was detected by quantitative RT-PCR. **d** Detection of Ezh2 binding to the *Bcl6* promoter. WT CD45.2^+^ T_FH_ and T_H_1 cells were sorted from recipient spleens on 5 *dpi*, and together with WT naïve CD4^+^ T cells, were analyzed by ChIP with anti-Ezh2 antibody or control IgG. Enriched Ezh2 binding at the TSSs of *Bcl6* or *Hprt1* genes was determined by ChIP-qPCR. Data in **c** and **d** are means ± s.d. from ≥2 experiments. **e**–**f** Impact of forced expression of Bcl6 on T_FH_ and antibody responses. WT or *Ezh2*^–/–^ Smarta CD4^+^ T cells were primed in vivo for 24 h and infected with empty vector (*EV*)-mCherry or *Bcl6*-mCherry retrovirus. Transduced Smarta CD4^+^ T cells were adoptively transferred into congenic mice, followed by LCMV-Arm infection (**e**), or into *Bcl6*^–/–^ recipients, followed by KLH-GP61 immunization (**f**). In **e**, mCherry^+^CXCR5^+^ SLAM^lo^ T_FH_ cells were detected in the recipient spleens on 3 dpi (corresponding to day 5 after initial priming), with representative contour plots and cumulative data on frequency and numbers of mCherry^+^CXCR5^+^SLAM^lo^ T_FH_ cells shown. In **f**, KLH-specific IgG was detected in the recipient sera on day 8 post-immunization. All cumulative data are means ± s.d. *p < 0.05; **p < 0.01; ***p < 0.001 for indicated pairwise comparison by Student’s *t*-test, coupled with one-way ANOVA for multi-group comparisons

Bcl6 is induced on all T_FH_ cells during an early lineage-specification stage and is maintained at a higher level in GC-T_FH_ cells^5,6^. To capture the early impact of Ezh2 deficiency on Bcl6 induction and T_FH_ cell differentiation and avoid potential secondary effects associated with increased apoptosis, we focused on 4 dpi, before CXCR5^+^PD-1^hi^ GC-T_FH_ cells are formed. At this timepoint, *Ezh2*^–/–^ Smarta CD4^+^ T cells exhibited marked reduction in CXCR5^+^SLAM^lo^ T_FH_ cells, accompanied by diminished Bcl6 expression and CXCR5^+^Bcl6^+^ T_FH_ cells (Fig. 3a, b). We further validated that *Bcl6* transcripts were diminished in early *Ezh2*^–/–^ Smarta T_FH_ cells (Fig. 3c) and that Ezh2 was directly bound to the *Bcl6* promoter in naïve CD4^+^ T cells, and the binding was enhanced in Smarta T_FH_ cells, but diminished in Smarta T_H_1 cells (Fig. 3d). These data substantiate the assertion that Ezh2 plays a critical role in Bcl6 induction.

We then tested the impact of forced Bcl6 expression by transducing in vivo primed WT or *Ezh2*^–/–^ Smarta CD4^+^ T cells with a bicistronic pMSCV-IRES-mCherry retrovirus, where mCherry marks retrovirally infected cells. Compared with empty-vector retrovirus that expressed mCherry alone (*EV*-mCherry), the Bcl6-expressing retrovirus (*Bcl6*-mCherry) directed most WT Smarta CD4^+^ cells to the T_FH_ lineage (Fig. 3e). In contrast, *EV*-mCherry-infected *Ezh2*^–/–^ Smarta CD4^+^ T cells remained defective in generation of CXCR5^+^ T_FH_ cells, while *Bcl6*-mCherry retrovirus promoted differentiation of *Ezh2*^–/–^ Smarta CD4^+^ cells to a T_FH_ fate (Fig. 3e). In addition, the number of *Ezh2*^–/–^ T_FH_ cells was partly restored by Bcl6, whereas that of *Ezh2*^–/–^ T_H_1 cells remained diminished (Fig. 3e). These observations support a specific role of the Ezh2-Bcl6 regulatory axis in T_FH_ lineage cells.

To test whether forced expression of Bcl6 in Ezh2-deficient T_FH_ cells could rectify B-cell helper function, CD45.1^+^CD4-Cre^+^*Bcl6*^fl/fl^ (*Bcl6*^–/–^) mice were used as adoptive-transfer recipients, in which the endogenous T_FH_ response was abrogated while B cells remained functional^5,39^. Retrovirus-infected Smarta CD4^+^ T cells were adoptively transferred into *Bcl6*^–/–^ recipients, which were then immunized with KLH-GP61. On day 8 post-immunization, KLH-specific IgG was detected in recipients of *EV*-mCherry-infected WT Smarta CD4^+^ cells, but was greatly diminished in *EV*-mCherry-infected *Ezh2*^–/–^ Smarta CD4^+^ cells (Fig. 3f). Recipients of *Bcl6*-mCherry-infected *Ezh2*^–/–^ Smarta CD4^+^ cells, however, showed greatly restored production of KLH-specific IgG (Fig. 3f). These data indicate that ectopic expression of Bcl6 functionally complements Ezh2 deficiency, strengthening the notion that Ezh2 acts upstream of Bcl6 to promote T_FH_ functional maturation.

### Ezh2 cooperates with Tcf1 to activate key T_FH_ genes

**Fig. 4:**
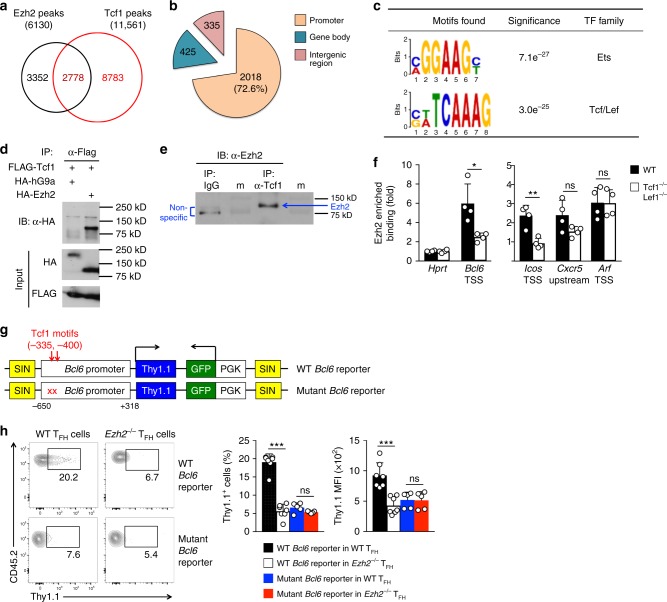
Ezh2 cooperates with Tcf1 to activate key T_FH_ genes. **a** Venn diagram showing overlap between Ezh2 and Tcf1 binding peaks in T_FH_ cells. **b** Distribution of Ezh2/Tcf1 co-occupied sites. *Promoter* is defined as +/–1 kb region flanking TSSs, and *gene body* refers to region covering +1 kb to transcription end site, with remainder as intergenic region. **c** De novo motif analysis of Ezh2/Tcf1 co-occupied sites. The motif logos and statistical significance are listed. **d** Ezh2, but not G9a, co-immunoprecipitates with Tcf1. FLAG-tagged Tcf1 was co-transfected with HA-tagged Ezh2 or G9a into 293T cells. The cell lysates were immunoprecipitated with anti-FLAG and immunoblotted with anti-HA. Data are representative of two experiments. **e** Ezh2 co-immunoprecipitates with Tcf1 in primary T_FH_ cells. CXCR5^+^SLAM^lo^ T_FH_ cells were sorted from WT mice infected with LCMV-Arm and immunoprecipitated with normal rabbit IgG or anti-Tcf1 followed by immunoblotting with anti-Ezh2. ‘m’ denotes marker lane. Data are representative of two experiments. **f** Ezh2 is recruited by Tcf1. WT or Tcf1^–/–^Lef1^–/–^ mice were infected with VacV, and CXCR5^+^SLAM^lo^ T_FH_ cells were sorted on 6 *dpi* and analyzed by ChIP with anti-Ezh2 antibody or control IgG. Enriched Ezh2 binding at the TSSs of *Bcl6*, *Icos*, *Arf* and a *Cxcr5* upstream region was determined by ChIP-qPCR. Data are means ± s.d. from two experiments with each sample measured in duplicates. ns, not statistically significant; *p < 0.05; **p < 0.01 by Student’s *t*-test. **g** Diagram of dual reporter retroviral construct. Red “x” denotes Tcf1 motif mutations in mutant *Bcl6* reporter. **h** Thy1.1 reporter expression driven by the *Bcl6* promoter. In vivo primed WT or *Ezh2*^–/–^ Smarta CD4^+^ T cells were infected with WT or mutant *Bcl6* reporter retrovirus, followed by adoptive transfer and LCMV-Arm infection. On 4 dpi (equivalent to day 6 after initial priming), GFP^+^CD45.2^+^ T_FH_ cells were detected in the recipient spleens and assessed for Thy1.1 expression. Cumulative data are means ± s.d. from experiments with ≥5 recipients analyzed for each genotype. ***p < 0.001 by Student’s *t*-test

Ezh2 does not bind DNA directly and is recruited to target gene loci by TFs. Tcf1 and its homolog Lef1 are critical for Bcl6 induction in T_FH_ cells^7–9^. By GSEA, gene sets containing Tcf1-activated genes in T_FH_ and GC-T_FH_ cells exhibited strong negative enrichment in *Ezh2*^–/–^ T_FH_ cells (Supplementary Figure 4a, b), suggesting Ezh2 and Tcf1 activate a common subset of target genes in the T_FH_ program. In T_FH_ cells, Tcf1 ChIP-Seq identified 11,561 Tcf1 binding peaks. Significantly, about 45% of Ezh2 peaks overlapped with Tcf1 peaks (Figs. 2e, 4a), and over 70% of these Ezh2/Tcf1 co-occupied regions were at gene promoters (Fig. 4b). By focusing on Ezh2 and Tcf1 peaks within +/–10 kb of TSSs, 2,219 Ezh2/Tcf1 peaks (group 1 in Fig. 2e) were enriched for Ets and Tcf/Lef consensus motifs (Fig. 4c). These Ezh2/Tcf1 peaks were associated with 2,975 unique Refseq genes. GSEA showed that the Ezh2/Tcf1 co-bound gene set was negatively enriched in *Ezh2*^–/–^ T_FH_ cells (Supplementary Figure 4c), corroborating the notion that Ezh2 and Tcf1 activate common target genes in the T_FH_ program.

Among the Ezh2/Tcf1-cobound genes downregulated in *Ezh2*^–/–^ T_FH_ cells were key T_FH_ target genes, such as *Bcl6*, *Icos*, and *Cxcr5* (Fig. 2g, Supplementary Figure 2d), suggesting Tcf1 recruits Ezh2 to these gene loci to exert activating function. HA-tagged Ezh2, but not HA-tagged G9a (another HMT that catalyzes H3K9 methylation) co-immunoprecipitated with FLAG-tagged Tcf1 (Fig. 4d). We validated co-immunoprecipitation of Ezh2 and Tcf1 in primary T_FH_ cells (Fig. 4e). We infected WT or Tcf1/Lef1-deficient mice (where both genes were specifically ablated in mature T cells^7^) with VacV, and performed ChIP of Ezh2 on the sorted T_FH_ cells. The enriched binding of Ezh2 to *Bcl6* and *Icos* TSSs was greatly diminished in Tcf1/Lef1-deficient T_FH_ cells, whereas Ezh2 binding to the *Cxcr5* upstream region was not affected (Fig. 4f). These data demonstrate that Ezh2 recruitment depends on Tcf1 at select, critical T_FH_ gene loci.

To further demonstrate direct regulation of Bcl6 by Ezh2, we employed an in vivo dual reporter assay, in which Thy1.1 and GFP reporters are embedded in a self-inactivated (SIN) retroviral vector, with phosphoglycerate kinase promoter (PGK)-driven GFP expression marking retrovirally transduced cells and Thy1.1 expression reporting *Bcl6* promoter activity (Fig. 4g)^40^. The Bcl6 promoter (–650 to +318 bp) depends on two adjacent Tcf1 motifs to activate the Thy1.1 reporter^9^. We retrovirally introduced the WT dual reporters into WT or *Ezh2*^–/–^ Smarta cells and performed adoptive transfer and LCMV-Arm infection. On 4 *dpi*, WT *Bcl6* promoter-driven Thy1.1 was expressed in WT GFP^+^ T_FH_ cells; in contrast, fewer *Ezh2*^–/–^ GFP^+^ T_FH_ cells expressed Thy1.1 and with greatly reduced gMFI (Fig. 4h). Consistent with previous reports^9^, mutating Tcf1 motifs in the *Bcl6* promoter similarly diminished Thy1.1 expression in WT GFP^+^ T_FH_ cells; however, the reduction of Thy1.1 expression in the mutant *Bcl6* reporter was not further exacerbated in *Ezh2*^–/–^ GFP^+^ T_FH_ cells (Fig. 4h). The latter observation further corroborates that Tcf1 and Ezh2 cooperatively activate *Bcl6* gene transcription. In addition, specific ablation of either Tcf1 or Ezh2 in mature CD4^+^ T cells impaired T_FH_ differentiation, and targeting both proteins almost completely abrogated the T_FH_ cell formation and Bcl6 induction elicited by viral infection (Supplementary Figure 4d). These data further support that Ezh2 and Tcf1 share common targets in activating the T_FH_ program, and suggest they have an additive effect in controlling distinct aspects of T_FH_ differentiation. These findings collectively identify Ezh2-mediated Bcl6 induction as a key regulatory axis in the generation of functionally competent T_FH_ cells.

### Ezh2 represses p19Arf to promote T_FH_ cell survival

**Fig. 5:**
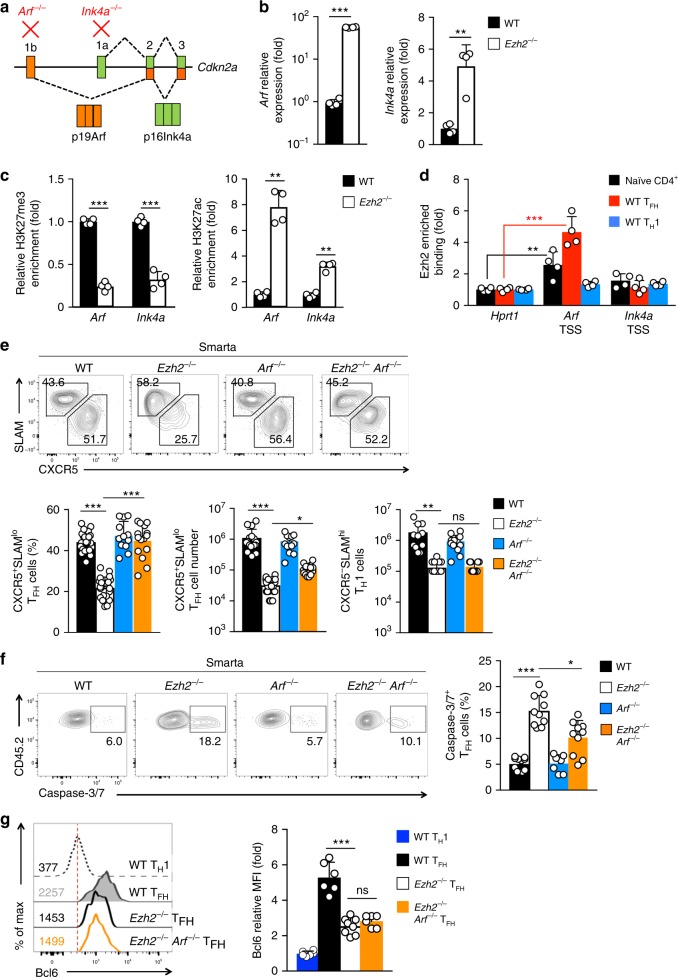
Ezh2 promotes T_FH_ differentiation by epigenetically repressing *Cdkn2a*. **a** *Cdkn2a* gene structure. The distal exon 1b and proximal exon 1a are differentially spliced with downstream exons to generate p19Arf and p16Ink4a, respectively. Targeting different exons (marked in red) achieves p19Arf- or p16Ink4a-specific deletion. **b** Detection of *Arf* and *Ink4a* transcripts in T_FH_ cells. CD45.2^+^ WT or *Ezh2*^–/–^ Smarta CD4^+^ T cells were adoptively transferred into congenic mice followed by infection with LCMV-Arm. On 4 *dpi*, CD45.2^+^ CXCR5^+^ SLAM^lo^ T_FH_ cells were sorted from the recipient spleens, and *Arf* and *Ink4a* transcripts from the *Cdkn2a* locus were detected by quantitative RT-PCR. **c** Detection of histone modification changes in T_FH_ cells. WT or *Ezh2*^–/–^ CD45.2^+^ CXCR5^+^ SLAM^lo^ T_FH_ cells were sorted on 4 *dpi* as in (**b**), and H3K27me3 and H3K27ac at the TSS of *Arf* and *Ink4a* were analyzed by ChIP-qPCR. **d** Detection of Ezh2 binding to the *Cdkn2a* locus. WT CD45.2^+^ T_FH_ and T_H_1 cells were sorted from the recipient spleens on 5 dpi as in (**b**), and together with WT naive CD4^+^ T cells, were subjected to ChIP analysis with anti-Ezh2 antibody or control IgG, and enriched Ezh2 binding at the TSS of *Arf* and *Ink4a* was determined by ChIP-qPCR. Data in (**b**–**d**) are means ± s.d. from ≥2 experiments, with each sample measured in duplicates. **e**–**g** Effect of genetically ablating p19Arf on rectifying T_FH_ defects due to Ezh2 deficiency. CD45.2^+^ Smarta CD4^+^ T cells from WT, *Ezh2*^–/–^, *Arf*^–/–^, or *Ezh2*^–/–^*Arf*^–/–^ Smarta-Tg mice were adoptively transferred into congenic mice followed by infection with LCMV-Arm. On 4 dpi, CXCR5^+^SLAM^lo^ T_FH_ cells were detected in the recipient spleens (**e**), and further analyzed for caspase-3/7 activation (**f**) and Bcl6 expression (**g**). CXCR5^–^SLAM^hi^ T_H_1 cells were analyzed for cell numbers (**e**) and Bcl6 expression (marked with dotted red line in **g**) for direct comparison with T_FH_ cells. Data are means ± s.d. from ≥2 experiments. *p < 0.05; **p < 0.01; ***p < 0.001 for indicated pairwise comparison by Student’s *t*-test, coupled with one-way ANOVA for multi-group comparisons

We next tested whether the conventional, HMT-dependent function of Ezh2 also contributed to T_FH_ cell differentiation. In T_FH_ cells, a small fraction of Ezh2 peaks were associated with strong H3K27me3 signals (Fig. 2e), so we cross-referenced the H3K27me3-associated Ezh2 peaks with genes upregulated in *Ezh2*^–/–^ T_FH_ cells (Fig. 2a, b). Our attention was directed to the *Cdkn2a* gene, a known Ezh2-repressed target in pancreatic β-cells and hematopoietic progenitors^41,42^. By alternative splicing, the *Cdkn2a* gene locus encodes two distinct proteins: 16 kDa Ink4a from a proximal promoter and 19 kDa Arf from a distal promoter (Fig. 5a). Whereas p16Ink4a inhibits cyclin-dependent kinases, p19Arf indirectly stabilizes p53; and both proteins regulate cell-cycle progression and apoptosis^43,44^.

Both *Arf* and *Ink4a* transcripts were highly induced in *Ezh2*^–/–^ Smarta T_FH_ cells in the adoptive transfer/LCMV-Arm infection model (Fig. 5b). In polyclonal WT T_FH_ cells, the *Cdkn2a* locus was marked with strong H3K27me3 but was devoid of H3K27ac (Supplementary Figure 5a). In WT Smarta T_FH_ cells, the *Arf* and *Ink4a* TSSs were marked by H3K27me3 (as detected by ChIP-PCR), while in Ezh2^–/–^ Smarta T_FH_ cells, H3K27me3 signals were not only markedly reduced but were also replaced by elevated H3K27ac signals (Fig. 5c). The Ezh2 binding pattern at the *Cdkn2a* locus appeared to be more spread than those at T_FH_ gene loci on Ezh2 ChIP-Seq (Supplementary Figure 5a, compare with Fig. 2g and Supplementary Figure 2d). Using site-specific ChIP-PCR, we found Ezh2 directly bound to the *Arf* TSS in naive CD4^+^ T cells, with the signal strengthened in Smarta T_FH_ but lost in Smarta T_H_1 cells (Fig. 5d). On the other hand, Ezh2 was not associated with *Ink4a* TSS in these CD4^+^ T cell subsets (Fig. 5d). In addition, the binding of Ezh2 to the *Arf* TSS did not depend on Tcf1 and Lef1 (Fig. 4f), consistent with the lack of Tcf1 peaks at this gene locus (Supplementary Figure 5a). These data indicate that the *Cdkn2a* locus is directly repressed by H3K27me3 deployed by Ezh2 HMT activity.

To determine if Ezh2-mediated repression of p19Arf or p16Ink4a was functionally critical for promoting T_FH_ differentiation, we generated *CD4-Cre*^+^*Arf*^FL/FL^ (*Arf*^–/–^) mice, specifically ablating p19Arf but leaving p16Ink4a intact^45^, and then generated Smarta *Arf*^–/–^ and Smarta *Ezh2*^–/–^*Arf*^–/–^ mice. Compound deletion of Ezh2 and p19Arf did not cause aberrant activation of CD4^+^ T cells (Supplementary Figure 5b). Deleting p19Arf alone did not exhibit detectably impact on T_FH_ cells elicited by the LCMV-Arm infection, and ablating p19Arf in *Ezh2*^–/–^ cells rectified the frequency of CXCR5^+^SLAM^lo^ T_FH_ cells and partially restored T_FH_ cell numbers on 4 dpi (Fig. 5e). Loss of Ezh2 caused increased apoptosis in Smarta T_FH_ cells, which was rectified by compound deletion of p19Arf (Fig. 5f). However, loss of p19Arf did not restore the number or survival defects in *Ezh2*^–/–^ T_H_1 cells (Fig. 5e, Supplementary Figure 5c), highlighting a T_FH_-specific effect. In addition, among *Ezh2*^–/–^ and *Ezh2*^–/–^*Arf*^–/–^ T_FH_ cells, the decrease in Bcl6 expression was similar (Fig. 5g). This result suggests that improved T_FH_ differentiation in *Ezh2*^–/–^*Arf*^–/–^ cells was not due to restored Bcl6 expression per se, implying that Ezh2-dependent Bcl6 induction and p19Arf repression are independent molecular events in T_FH_ cells.

To ablate p16Ink4a specifically but leave p19Arf intact, we also generated Smarta *CD4-Cre*^+^*Ink4a*^FL/FL^ (Smarta *Ink4a*^–/–^) mice^46^. Compound deletion of Ezh2 and p16Ink4a did not detectably perturb CD4^+^ T cell homeostasis (Supplementary Figure 5b), and did not detectably ‘rescue’ *Ezh2*^–/–^ T_FH_ frequency, numbers, or Bcl6 expression (Supplementary Figure 5d,e). In addition, Ezh2 deficiency compromised accumulation of effector CD8^+^ T cells, in line with published observations^47–49^, a defect not rectified by compound deletion of p19Arf (Supplementary Figure 5f). These data collectively indicate that Ezh2-mediated repression of p19Arf is an important and specific regulatory axis required for promoting T_FH_ differentiation and survival.

### Ezh2 represses Arf to prevent antagonizing Bcl6 function

**Fig. 6:**
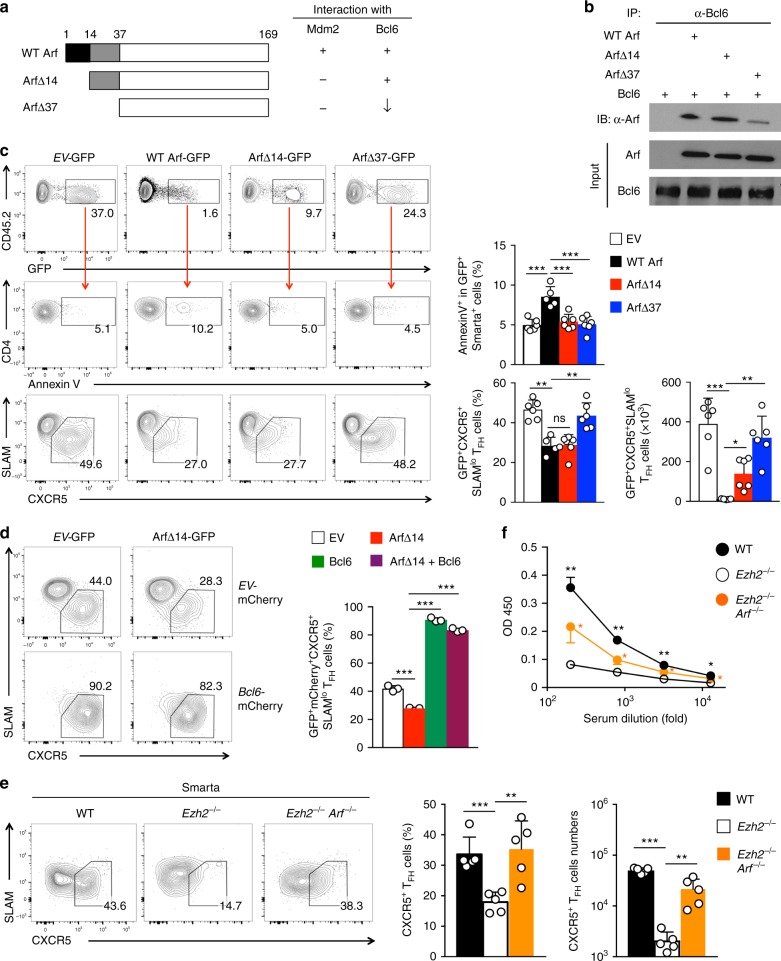
Aberrantly upregulated p19Arf antagonizes T_FH_ differentiation and survival. **a** Diagram showing the structure of WT and mutant forms of p19Arf, with their capacity of interacting with Mdm2 and Bcl6 summarized. **b** p19Arf and Bcl6 interaction capacity. WT or mutant p19Arf was co-transfected with WT Bcl6 expression vector into 293T cells. Cell lysates were immunoprecipitated with anti-Bcl6 and then immunoblotted with anti-p19Arf. Data are representative of two experiments. **c** Impact of p19Arf on T_FH_ differentiation and survival. In vivo primed WT Smarta CD4^+^ T cells were infected with *EV*-GFP retrovirus or that expressing WT or mutant p19Arf, followed by adoptive-transfer and LCMV-Arm infection. On 6 dpi (corresponding to day 5 after initial priming), CD45.2^+^CD4^+^ T cells were detected for GFP expression (top panels), and GFP^+^ cells were analyzed for AnnexinV positivity (middle) or CXCR5^+^SLAM^lo^ T_FH_ cells (bottom panels). **d** Interplay of p19Arf and Bcl6 in T_FH_ differentiation. WT Smarta CD4^+^ T cells were transduced with *EV-*GFP or ArfΔ14 retrovirus in combination with *EV*-mCherry or Bcl6 retrovirus, followed by adoptive-transfer and LCMV-Arm infection. On 6 dpi, GFP^+^mCherry^+^CD45.2^+^ CD4^+^ T cells were analyzed for frequency of CXCR5^+^SLAM^lo^ T_FH_ cells. **e**–**f** Effect of genetically ablating p19Arf on T_FH_ and B cell responses to protein immunization. WT, *Ezh2*^–/–^, or *Ezh2*^–/–^*Arf*^–/–^ CD45.2^+^ Smarta CD4^+^ T cells were adoptively transferred into CD45.1^+^*Bcl6*^–/–^ recipients followed by GP61-KLH immunization. On day 5 post-immunization, CXCR5^+^ T_FH_ cells were detected in the draining LNs (**e**), and on day 8, KLH-specific IgG was detected in the sera by ELISA (**f**). Data are in (**c**–**f**) are means ± s.d. from ≥2 experiments. *p < 0.05; **p < 0.01; ***p < 0.001 for indicated pairwise comparison (**c**–**e**) or comparison with recipients of *Ezh2*^–/–^ Smarta CD4^+^ T cells (**f**) by Student’s *t*-test, coupled with one-way ANOVA for multi-group comparisons

A conventional role for p19Arf is to interact with Mdm2, a negative regulator of p53, and induce apoptosis and/or cell-cycle arrest. The N-terminal 1–14 amino acids of p19Arf mediate interaction with Mdm2 (Fig. 6a)^50,51^. p19Arf is also reported to physically interact with Bcl6 through its N-terminal 1–37 amino acids (Fig. 6a), to perturb Bcl6-mediated gene repression, as assayed in vitro^52^. Therefore, the improved T_FH_ differentiation in *Ezh2*^–/–^*Arf*^–/–^ Smarta cells might not be solely ascribed to increased cell survival and/or expansion.

We then performed structure-function analysis, by generating two mutant forms of p19Arf, ArfΔ14 and ArfΔ37, which lacked N-terminal 14 and 37 amino acids, respectively. By co-immunoprecipitation, both WT p19Arf and ArfΔ14 interacted with Bcl6 similarly, whereas the ArfΔ37 mutant pulled down less Bcl6 (Fig. 6b). We next transduced WT CD45.2^+^ Smarta CD4^+^ T cells with a bicistronic pMSCV-IRES-GFP retrovirus expressing WT or mutant p19Arf protein, followed by adoptive transfer and LCMV-Arm infection. When examined on 6 dpi, GFP^+^ Smarta CD4^+^ cells infected with WT p19Arf-GFP retrovirus were detected at a substantially lower frequency than those infected with empty-vector retrovirus expressing GFP alone (*EV*-GFP). This was partly due to enhanced apoptosis, as measured by Annexin V positivity (Fig. 6c). Among the remaining WT p19Arf-expressing GFP^+^ Smarta cells, T_FH_ cells were greatly diminished in frequency and number, and CXCR5 expression was decreased (Fig. 6c), suggesting aberrantly induced p19Arf not only induced cell death but also impeded T_FH_ differentiation. ArfΔ14 abrogates the interaction with Mdm2 and prevents p53 induction^50,51^, and forced expression of ArfΔ14 in Smarta CD4^+^ T cells substantially alleviated cell death; however, ArfΔ14 could still impede T_FH_ differentiation (Fig. 6c, third column), likely because it could still interact with Bcl6 effectively. In contrast, ArfΔ37, which only weakly binds Bcl6 and does not interact with Mdm2, had little effect on inducing cell death or impairing T_FH_ differentiation (Fig. 6c, fourth column). These data indicate that aberrant p19Arf induction has a dual impact: it induces apoptosis/cell-cycle arrest and impedes T_FH_ differentiation. The latter is likely ascribed to an antagonistic effect on Bcl6 through protein–protein interaction.

To substantiate this point, we reasoned that forced expression of Bcl6 would counteract against p19Arf’s inhibitory effect. We then tested ArfΔ14, which retained the ability to interact with Bcl6 and inhibit T_FH_ differentiation without strongly inducing cell death/growth arrest (Fig. 6c). When Bcl6 was co-expressed with ArfΔ14, T_FH_ differentiation was substantially restored (Fig. 6d), indicating that elevated expression of Bcl6 was sufficient to overcome inhibition by p19Arf. Therefore, Ezh2 HMT-dependent repression of p19Arf is not only important for promoting cell survival/expansion, but also critical for preventing direct inhibition of Bcl6 activity.

The functional requirement for Ezh2-mediated p19Arf repression in T_FH_ cells was assessed by measuring their B-cell helper capacity. WT, *Ezh2*^–/–^, or *Ezh2*^–/–^*Arf*^–/–^ Smarta CD4^+^ T cells were adoptively transferred into CD45.1^+^*Bcl6*^–/–^ mice, which were then immunized with KLH-GP61. Loss of Ezh2 diminished differentiation of Smarta T_FH_ cells in response to protein immunization, and this defect was partially rectified if p19Arf was ablated together with Ezh2 (Fig. 6e). In addition, production of KLH-specific IgG in the *Bcl6*^–/–^ recipients was partly restored by compound deletion of p19Arf and Ezh2 (Fig. 6f). These data illustrate an essential requirement for Ezh2-dependent p19Arf repression in generating functionally competent T_FH_ cells.

### Ezh2 Ser21 phosphorylation is necessary for Bcl6 induction

**Fig. 7:**
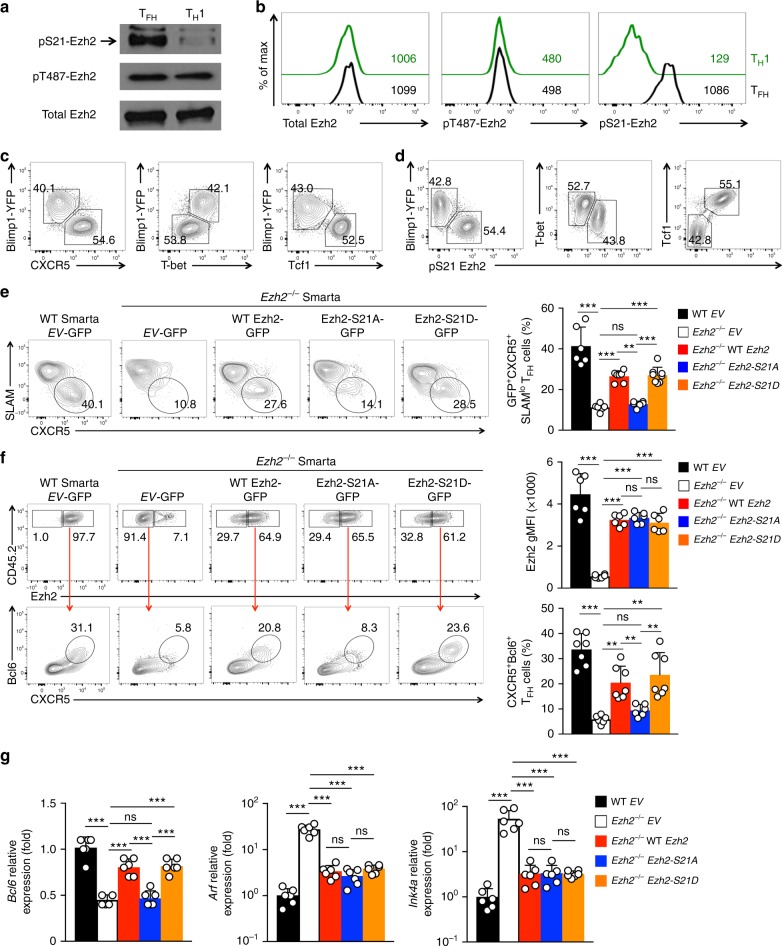
Ezh2 Ser21 phosphorylation is necessary for Bcl6 induction but dispensable for p19Arf repression. **a** Ezh2 phosphorylation status in polyclonal CD4^+^ T cell responses. CXCR5^+^SLAM^lo^ T_FH_ cells and CXCR5^–^SLAM^hi^ T_H_1 cells were sorted from activated CD4^+^ T cells in spleens of LCMV-Arm-infected WT C57BL/6 mice on 8 dpi, and immunoblotted with antibodies specific for pS21-Ezh2, pT487-Ezh2, and total Ezh2. Data are representative from 2 independent experiments with similar results. **b** Ezh2 phosphorylation status in monoclonal CD4^+^ T cell responses. CD45.2^+^ WT Smarta CD4^+^ T cells were adoptively transferred into congenic recipients followed by LCMV-Arm infection. On 4 dpi, CXCR5^+^SLAM^lo^ T_FH_ cells and CXCR5^–^SLAM^hi^ T_H_1 cells were analyzed for total Ezh2, pT487-Ezh2, or pS21-Ezh2 with intracellular staining. Values denote gMFI, and data are representative of ≥3 experiments. **c**–**d** Predominant association of pS21-Ezh2 with T_FH_ lineage cells. Blimp1-YFP^+^ Smarta CD4^+^ T cells were adoptively transferred into WT congenic recipients followed by LCMV infection. On 4 dpi, donor-derived CD4^+^ T cells were analyzed for Blimp1-YFP, CXCR5, T-bet, Tcf1 and pS21-Ezh2 at indicated combinations. **e**–**g** Effect of WT or mutant Ezh2 on rectifying T_FH_ defects. *Ezh2*^–/–^ Smarta CD4^+^ T cells were primed in vitro and transduced with *EV-*GFP retrovirus or that expressing WT or mutant forms of Ezh2, followed by adoptive transfer and LCMV-Arm infection. WT Smarta CD4^+^ cells infected with *EV-*GFP retrovirus were used as a control. On 4 dpi, equivalent to day 7 after initial CD4^+^ T cell priming, GFP^+^ Smarta CD4^+^ T cells were analyzed for frequency of CXCR5^+^SLAM^lo^ T_FH_ cells (**e**). For a more accurate detection of the CXCR5^+^Bcl6^+^ T_FH_ subset in retrovirally transduced cells, CD45.2^+^CD4^+^ T cells were intracellularly stained for Ezh2, and Ezh2^–^ cells in the *EV-*GFP group and Ezh2^+^ cells in other groups were analyzed (**f**). In (**g**), GFP^+^CD45.2^+^CXCR5^+^SLAM^–^ T_FH_ cells were sorted for analysis of *Bcl6*, *Arf*, and *Ink4a* transcripts by quantitative RT-PCR. Cumulative data are means ± s.d. from ≥2 independent experiments. ns, not statistically significant; *p < 0.05; **p < 0.01; ***p < 0.001 for indicated pairwise comparison by Student’s *t*-test, coupled with one-way ANOVA for multi-group comparisons

We next investigated how Ezh2 could adopt the dual functions of transcriptional coactivator and epigenetic silencer in T_FH_ cells. Previously it was shown that, in transformed cells, post-translational modification of Ezh2 (e.g., phosphorylation at Ser21 and Thr487^53,54^) contributes to a functional switch^35^. We then detected Ezh2 Ser21 or Thr487 phosphorylation (pS21-Ezh2 or pT487-Ezh2) status by immunoblotting of sorted polyclonal T_FH_ and T_H_1 cells elicited by LCMV-Arm infection. Whereas pT487-Ezh2 was similar in both cell types, pS21-Ezh2 was detected at a much higher level in T_FH_ than T_H_1 cells (Fig. 7a). In monoclonal Smarta CD4^+^ T cell responses, pS21-Ezh2 was predominantly detected in T_FH_ cells, whereas signals of total Ezh2 and pT487-Ezh2 were similar between CXCR5^+^SLAM^lo^ T_FH_ and CXCR5^–^SLAM^hi^ T_H_1 cells (Fig. 7b), demonstrating that Ezh2 Ser21 phosphorylation is predominantly associated with T_FH_ cells.

To substantiate the linkage between pS21-Ezh2 and T_FH_ cells beyond solely relying on cell surface markers, we measured the correlation of pS21-Ezh2 with T_H_1 and T_FH_-characteristic TFs. The combination of Blimp1-YFP reporter and CXCR5 surface staining clearly distinguished T_H_1 and T_FH_ cells elicited by LCMV infection^5,8^, and the Blimp1-YFP expression showed concordant expression with the T_H_1 lineage-defining T-bet, but a distinct pattern with the T_FH_-characteristic Tcf1 expression^7–9^ (Fig. 7c). In this context, pS21-Ezh2 was detected at much lower levels in Blimp1-YFP^hi^ or T-bet^hi^ T_H_1 cells, but at a much higher level in Tcf1^hi^ T_FH_ cells (Fig. 7d). These data indicate that pS21-Ezh2 is more strongly associated with the T_FH_ lineage than T_H_1 cells.

To determine if pS21-Ezh2 was functionally important for T_FH_ differentiation, we generated phosphorylation-resistant and phospho-mimetic Ezh2 mutants by replacing Ser21 with Ala (Ezh2-S21A) and with Asp (Ezh2-S21D), respectively. These Ezh2 proteins were then expressed in *Ezh2*^–/–^ Smarta CD4^+^ T cells by pMSCV-IRES-GFP retroviruses. Compared with *EV*-GFP retrovirus, forced expression of WT Ezh2, Ezh2-S21A, or Ezh2-S21D all resulted in increased expansion of *Ezh2*^–/–^ GFP^+^ Smarta CD4^+^ T cells (Supplementary Figure 6a). Forced expression of WT Ezh2 or the phospho-mimetic Ezh2-S21D in *Ezh2*^–/–^ cells showed significant increase in SLAM^lo^CXCR5^+^ T_FH_ cells among GFP^+^ transduced cells, whereas phosphorylation-resistant Ezh2-S21A had only a marginal effect, if any (Fig. 7e). Importantly, in the transduced cells, WT Ezh2 and both mutant Ezh2 proteins showed similar levels of expression (Fig. 7f, top panels). Compared with Ezh2-S21A, both WT Ezh2 and Ezh2-S21D were more effective in restoring generation of Bcl6^+^CXCR5^+^ T_FH_ cells in *Ezh2*^–/–^ cells (Fig. 7f, bottom panels), in inducing *Bcl6* transcripts and CXCR5 and ICOS protein expression in GFP^+^ *Ezh2*^–/–^ T_FH_ cells (Fig. 7g, Supplementary Figure 6b). These observations indicate that Ezh2 Ser21 phosphorylation is critical for optimal activation of *Bcl6* transcription and the T_FH_ program, whereas phosphorylation-resistant Ezh2 has limited capacity in this regard. It is also noteworthy, however, that both Ezh2-S21A and Ezh2-S21D were similar to WT Ezh2 in repressing aberrant upregulation of the *Arf* and *Ink4a* transcripts in GFP^+^ *Ezh2*^–/–^ T_FH_ cells (Fig. 7g), consistent with the observation that all Ezh2 forms, regardless of Ser21 phosphorylation status, had similar capacity in elevating expansion of *Ezh2*^–/–^ Smarta CD4^+^ T cells (Supplementary Figure 6a).

### Dual requirements for Ezh2 in T_FH_ lineage specification

**Fig. 8:**
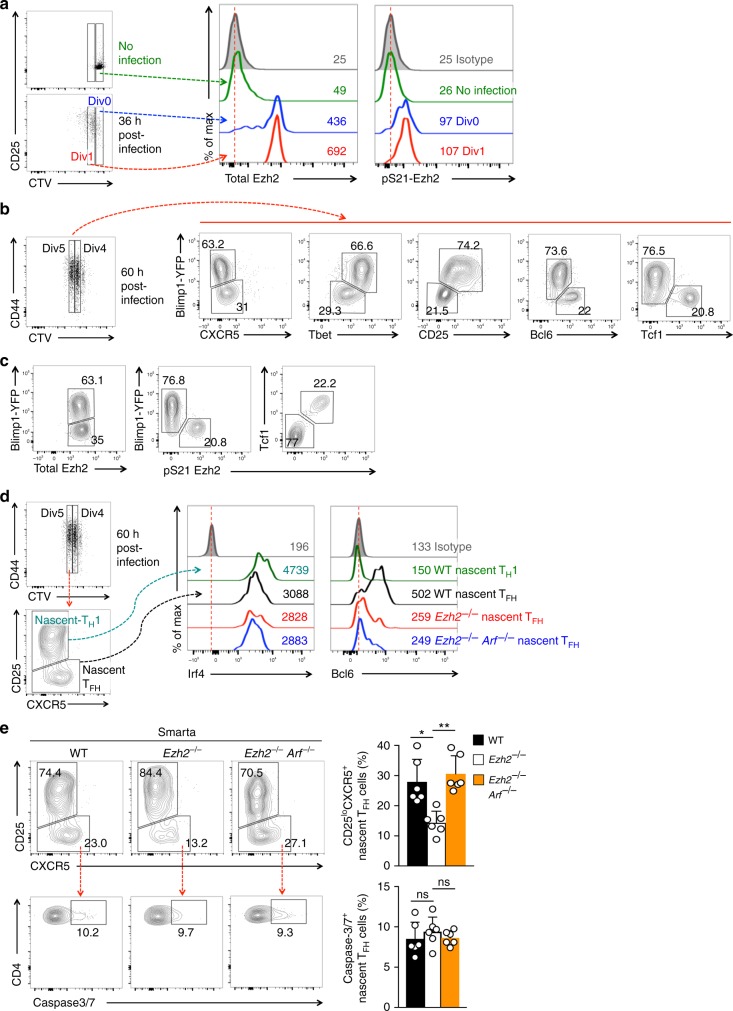
Ezh2 is necessary for T_FH_ lineage specification during the early activation stage. **a** Rapid induction of Ezh2 and Ser21 phosphorylation upon CD4^+^ T cell activation in vivo. CTV-labeled WT Smarta CD4^+^ T cells were adoptively transferred into B6.SJL recipients, either uninfected or infected with LCMV-Arm. Thirty-six hours later, undivided donor cells (Div0) or those in the first division (Div1) were analyzed for expression of CD25, total Ezh2 and pS21-Ezh2. Values denote geometric MFI, and data are representative from at least 2 independent experiments. **b**–**c** Predominant association of pS21-Ezh2 with nascent T_FH_ cells at the fate-bifurcation stage. CTV-labeled Blimp1-YFP^+^ Smarta CD4^+^ T cells were adoptively transferred into WT congenic recipients followed by LCMV infection. Sixty hours later, donor cells in the 5th division were analyzed for Blimp1-YFP in combination with surface staining of CXCR5 or CD25 or intracellular staining of T-bet and Tcf1 (**b**), or with total Ezh2 and pS21-Ezh2 (**c**). **d**–**e** Impact of Ezh2 deficiency on early T_FH_ cells. CTV-labeled WT, *Ezh2*^–/–^, or *Ezh2*^–/–^*Arf*^–/–^ Smarta CD4^+^ T cells were adoptively transferred, and recipients infected with LCMV-Arm as in (**b**). Cells in the 5th division at 60 h post-infection were analyzed for nascent T_FH_ and T_H_1 subsets based on CD25 and CXCR5 expression. The nascent T_FH_ and T_H_1 cells were then analyzed for expression of T_H_1-associated Irf4 and T_FH_-associated Bcl6 (**d**), with values denoting geometric MFI and dotted lines marking histogram peaks in isotype control staining. Caspase-3/7 activation was determined in nascent T_FH_ cells (lower panels in **e**). Cumulative data are means ± s.d. from ≥2 independent experiments. ns, not statistically significant; *p < 0.05; **p < 0.01 for indicated pairwise comparison by Student’s *t*-test, coupled with one-way ANOVA for multi-group comparisons

T_H_1 and T_FH_ bifurcation is believed to start during early division events, right after CD4^+^ T cell activation^5,8,55,56^. To test if Ezh2 is required at the early T_H_1-T_FH_ fate-bifurcation stage in vivo, WT Smarta CD4^+^ T cells were labeled with cell-trace violet (CTV) and adoptively transferred into congenic recipients, followed by LCMV-Arm infection. Thirty-six hours later, the Smarta CD4^+^ T cells were activated and initiated the first division, which was accompanied by elevated CD25 expression (Fig. 8a). Although Ezh2 expression was low in naïve Smarta CD4^+^ T cells (as seen in uninfected recipients), it was strongly induced after activation and proliferation (Fig. 8a). Importantly, Ser21 phosphorylation was detected on the induced Ezh2 protein in activated CD4^+^ T cells, even before the first division, and pS21-Ezh2 persisted in dividing cells (Fig. 8a).

At 60 h post-infection, the activated Smarta CD4^+^ T cells were mostly in the 4th and 5th divisions, a window in which nascent T_H_1 and T_FH_ cells emerge^5,8^, with nascent T_H_1 expressing higher Blimp1-YFP and nascent T_FH_ cells being Blimp1-YFP^lo^. Using adoptive transfer of CTV-labeled Blimp1-YFP^+^ Smarta CD4^+^ T cells followed by LCMV infection, we distinguished Blimp1-YFP^hi^ nascent T_H_1 and Blimp1-YFP^lo^ nascent T_FH_ cells, at the 5th division at 60 h post-infection (Fig. 8b). Blimp1-YFP^hi^ nascent T_H_1 cells expressed more CD25 and T-bet, but were Bcl6^–/lo^ and Tcf1^–/lo^ (Fig. 8b). On the other hand, although CXCR5 induction in the early stage Blimp1-YFP^lo^ nascent T_FH_ cells was not as distinguishable as in fully committed T_FH_ cells at a later time point, the Blimp1-YFP^lo^ cells expressed more Bcl6 and Tcf1, but were CD25^–/lo^ and T-bet^–/lo^, consistent with a T_FH_ fate (Fig. 8b). In this context, both nascent T_H_1 and T_FH_ cells expressed similar levels of total Ezh2; in contrast, pS21-Ezh2 was only detected in Blimp1-YFP^lo^ or Tcf1^hi^ nascent T_FH_ cells (Fig. 8c). This analysis demonstrates concordant association of Ezh2 Ser21 phosphorylation with Bcl6 induction and Tcf1 preservation at the early T_FH_ lineage specification stage.

To investigate a role of Ezh2 in T_FH_ lineage specification, CTV-labeled WT or *Ezh2*^–/–^ Smarta CD4^+^ T cells were adoptively transferred, followed by LCMV-Arm infection. At 45 h post-infection, Smarta CD4^+^ T cells of both genotypes underwent two to three divisions, divided at similar rates, and showed similar levels of CD44 induction (Supplementary Figure 7a). Similar results were observed at 60 h post-infection, when both WT and *Ezh2*^–/–^ Smarta CD4^+^ T cells were in 4th and 5th divisions (Supplementary Figure 7a). At 60 h post-infection, *Ezh2*^–/–^ Smarta CD4^+^ T cells were detected in recipient spleens in similar numbers as WT cells, and showed similar rate of Caspase-3/7 activation (Supplementary Figure 7b, c). These data indicate that loss of Ezh2 did not perturb CD4^+^ T cell activation and early division, consistent with previous reports^29,30^. This is in contrast to the profound reduction of *Ezh2*^–/–^ Smarta CD4^+^ T cells at 4 dpi (Supplementry Fig. 3a), and thus provides a time window where CD4^+^ T cell survival/expansion is not affected by Ezh2 deficiency and allows for characterization of T_H_1 versus T_FH_ lineage bifurcation. Because of the concordant expression of Blimp1-YFP with CD25 (ref. ^5^ and Fig. 8b), the combination of CD25 and CXCR5 was used to identify nascent T_H_1 and T_FH_ cells in activated CD4^+^ T cells. Although CXCR5 expression was not strong, CD25^hi^CXCR5^–^ cells expressed more Irf4^15,57^, and CD25^lo^CXCR5^+/lo^ cells predominantly expressed Bcl6 (Fig. 8d), validating CD25 and CXCR5 surface marker-based distinction of nascent T_H_1 and T_FH_ cells. In the 5th division, *Ezh2*^–/–^ nascent T_FH_ cells were detected at a frequency far lower than that of their WT counterparts (Fig. 8e). In addition, *Ezh2*^–/–^ nascent T_FH_ cells expressed substantially less Bcl6 than WT cells, with no increase in T_H_1-associated Irf4 expression (Fig. 8d). These observations indicate that Ezh2 expression, which is predominantly in a Ser21 phosphorylated form in nascent T_FH_ cells (Fig. 8c), is critical for inducing Bcl6 at the very early stage of T_H_1-T_FH_ fate bifurcation.

To determine if p19Arf repression by Ezh2 HMT contributes to the early T_FH_ lineage specification, we analyzed the behavior of adoptively transferred, CTV-labeled *Ezh2*^–/–^*Arf*^–/–^ Smarta CD4^+^ T cells in response to LCMV-Arm infection. Similar to WT and *Ezh2*^–/–^ cells, *Ezh2*^–/–^*Arf*^–/–^ Smarta CD4^+^ T cells did not exhibit defects in early activation, division, cell accumulation or survival (Supplementary Figure 7a–c). Strikingly, however, the reduction of nascent T_FH_ frequency observed in *Ezh2*^–/–^ cells was rectified by ablating *Arf* (Fig. 8e). In *Ezh2*^–/–^*Arf*^–/–^ nascent T_FH_ cells, Bcl6 expression remained as low as that in *Ezh2*^–/–^ cells (Fig. 8d), and cell survival was not detectably different among WT, *Ezh2*^–/–^ and *Ezh2*^–/–^*Arf*^–/–^ cells (Fig. 8e, lower panels). These data, coupled with the observations that abnormally upregulated p19Arf antagonized Bcl6 activity and impeding T_FH_ differentiation (Fig. 6), prompt us to posit that lifting the Bcl6-antagonistic effect driven by p19Arf is sufficient to ‘rescue’ T_FH_ lineage specification, even if Bcl6 induction *per se* is suboptimal in the absence of Ezh2. Thus, Ezh2 is critical for specifying activated CD4^+^ T cells to the T_FH_ lineage, and does so through two parallel mechanisms: by transcriptionally activating Bcl6 in its Ser21 phosphorylated form and by epigenetically silencing Arf, to prevent an antagonistic effect on the induced Bcl6.

## Discussion

In this study, we show Ezh2 acts early in T_FH_ lineage specification stage and utilizes multipronged mechanisms to promote production of functionally competent T_FH_ cells. Since Ezh2 HMT activity can catalyze H3K27me3, its regulatory roles are largely ascribed to direct repression of target genes^58,59^. Ezh2 is indeed responsible for repression of alternative helper lineage-associated genes when CD4^+^ T cells are polarized in vitro, *i*.*e*., repressing the expression of Gata3 and IL-4 in T_H_1 cells and that of T-bet and IFN-γ in T_H_2 cells^28,29,60^. In Treg cells, Ezh2 is recruited by Foxp3 to enforce gene repression and hence maintain a Treg cell identity upon activation^32,33^. In T_FH_ cells, however, global mapping of Ezh2 occupancy and H3K27 modification status revealed that Ezh2 is predominantly associated with H3K27ac rather than H3K27me3. In addition, ablating Ezh2 in T_FH_ cells downregulated more genes than it upregulated, especially genes associated with the T_FH_ transcription program. Although we cannot completely rule out a secondary effect, such as derepression of transcriptional/epigenetic repressor(s), our data strongly support a unique function of Ezh2 in transcriptional activation of the T_FH_ program.

How does Ezh2 switch from an epigenetic repressor to a transcriptional activator in the context of T_FH_ cells? Several factors might contribute. The first lies with post-translational modification of Ezh2, in particular Ser21 phosphorylation. We showed that pS21-Ezh2 is predominantly associated with T_FH_ cells, from nascent T_FH_ cells at the lineage-specification stage to fully committed T_FH_ cells after functional maturation. Significantly, pS21-Ezh2 is functionally important in transactivating Bcl6 and the T_FH_ program, revealing a critical contribution by Ezh2 phosphorylation to a non-cancerous, physiological process. As for kinase(s) that phosphorylate Ezh2 Ser21, the PI3K-Akt pathway might be a candidate, based on previous studies in cell lines^35,54^. It has been reported that mTOR and Akt pathways are necessary to promote T_FH_ differentiation^61,62^, although Akt appears to be more active in T_H_1 cells^55,63^. During T_FH_ differentiation, Akt activity can be more dynamically regulated in strength and duration by costimulatory pathways during T_FH_ differentiation, further studies are therefore needed to delineate signaling kinetics that leads to the predominant association of pS21-Ezh2 with T_FH_ cells.

The second contributing factor might be the availability of key transcription factor(s) that recruit Ezh2 to target gene loci. Using systems biology approaches, we found enriched Tcf/Lef motifs in Ezh2 peaks and substantial co-occupancy of Ezh2 and Tcf1 in the broad T_FH_ program. During in vivo responses to acute viral or bacterial infections, Tcf1 expression is specifically retained in T_FH_ cells, and at a level similar to naïve T cells, while in T_H_1 cells Tcf1 is drastically downregulated^7,9^. The presence of Tcf1 at T_FH_ target gene loci such as *Bcl6* allows recruitment of pS21-Ezh2 to enhance transcriptional activation. β-catenin is a known Tcf1 coactivator^64^, but the Tcf1-β-catenin interaction appears to be dispensable for Bcl6 expression in T_FH_ cells^65^. Our data further suggest Tcf1 utilizes Ezh2 as a coactivator instead of β-catenin in T_FH_ cells. Compound deletion of both Tcf1 and Ezh2 further exacerbated T_FH_ defects, suggesting each factor controls distinct aspects of the T_FH_ program, in addition to their shared regulatory targets. For example, Blimp1 was upregulated in Tcf1-deficient T_FH_ cells^7,8^ but was unaffected by Ezh2 ablation; in contrast, *Cdkn2a* was induced in Ezh2-deficient T_FH_ cells but not affected by Tcf1 ablation. Notably, Tcf1 is by no means the sole TF recruiting Ezh2 to activate the T_FH_ program, because binding to a *Cxcr5* upstream regulatory region did not appear to depend on Tcf1, and Ezh2 peaks were enriched for the Ets motif, which could be utilized by any of >30 Ets family members.

Ezh2 is required for survival of diverse cell types and, in the case of lymphocytes, is necessary for clonal expansion in response to stimulation of the antigen receptors. In the literature, it is frequently described that Ezh2-deficient T cells fail to accumulate during immune responses to infections or tumor antigen^47–49^. Similarly, we observed greatly diminished numbers of Ezh2-deficient T_FH_ cells, starting from day 4 post-infection, at least partly owing to increased apoptosis. Analyses of Ezh2 downstream genes identified *Cdkn2a*, whose repression depended on the conventional Ezh2 HMT activity. By genetic ablation of the p19Arf product from the *Cdkn2a* locus, the enhanced apoptosis in Ezh2-deficient T_FH_ cells was alleviated, leading to at least partial restoration of T_FH_ cell accumulation in the absence of Ezh2. It is of note that Ezh2-mediated p19Arf repression appears to function specifically in T_FH_ cells since dual targeting of p19Arf and Ezh2 did not boost accumulation of Ezh2-deficient T_H_1 or effector CD8^+^ T cells in response to viral infection. In line with this view, in GC B cells, Ezh2 epigenetically silences *Cdkn1a*, which encodes another cyclin-dependent kinase inhibitor (p21Cip1), to control cell-cycle progression^66^. Therefore, a universal requirement for Ezh2 in cell survival and growth may utilize distinct mechanisms depending on cell context, which merits a case-by-case investigation.

Our studies reveal that Ezh2-mediated repression of p19Arf also played an unconventional role, beyond promoting T_FH_ cell survival and expansion. Built on a previous in vitro characterization of p19Arf-Bcl6 interaction^52^, we dissociated the p53-inducing function of p19Arf from its ability to interact with Bcl6 through structural dissection. We demonstrated that the derepressed p19Arf impeded T_FH_ differentiation in vivo, which was neutralized/rectified by forced expression of Bcl6. Furthermore, compared with *Ezh2*^–/–^ T_FH_ cells, *Arf*^–/–^*Ezh2*^–/–^ T_FH_ cells were improved in, not only survival and accumulation but also B cell-help function. These findings reveal a specific function for p19Arf in antagonizing Bcl6 activity in T_FH_ cells in vivo, which is weaved into its known role in cell survival/expansion.

In addition to genetic complementation approaches, we carefully examined the kinetics of CD4^+^ T cell responses and the involvement of Ezh2. Following in vivo stimulation by viral infection, Ezh2 was potently induced after CD4^+^ T cell activation, even before cell division, similar to the induction of CD25. This places Ezh2 among the early response genes in response to TCR stimulation. Within 60 h of CD4^+^ T cell activation when they reached the 4th and 5th divisions, the loss of Ezh2 did not affect T cell activation, survival, early division and cell accumulation. Within this ‘survival-intact’ window, we demonstrated that Ezh2 specifies the activated CD4^+^ T cells to the T_FH_ lineage, further dissociating a T_FH_-specific regulatory function from its ‘universal’ pro-survival role. Notably, pS21-Ezh2 was predominantly associated with nascent T_FH_ cells, and was necessary for optimal induction of Bcl6 in further committed T_FH_ cells. Interestingly, the reduced frequency of early T_FH_ cells caused by Ezh2 deficiency was rectified by a compound deletion with p19Arf, whereas Bcl6 expression remained suboptimal. This observation highlights the necessity of lifting the Bcl6-antagonistic effect by aberrantly elevated p19Arf. We therefore posit that, from the early stage of CD4^+^ T cell activation, Ezh2 Ser21 phosphorylation-dependent Bcl6 activation and Ezh2 HMT-dependent p19Arf repression are already in place and actively contribute to T_FH_ lineage specification.

In summary, our studies demonstrate the essential roles of Ezh2 in promoting T_FH_ differentiation and functional maturation, to help mount protective antibody responses. To meet these functional requirements, Ezh2 employs multipronged mechanisms. One involves its known polycomb-dependent HMT activity to repress the expression of p19Arf from the *Cdkn2a* locus, and thus promotes T_FH_ lineage commitment and functional maturation by shielding T_FH_ cells from Bcl6 inhibition and promotes T_FH_ cell survival by shielding them from p53 induction. Unexpectedly, a more prominent role of Ezh2 in T_FH_ cells is associated with broad activation of genes in the T_FH_ transcriptional program. Significantly, Bcl6, the T_FH_ lineage-defining TF, is a major Ezh2 downstream target, and Ezh2-mediated Bcl6 transcriptional activation depends on phosphorylation of Ezh2 at Ser21 and recruitment of Ezh2 by Tcf1 to the *Bcl6* gene locus. These findings demonstrate that Ezh2 has a heretofore unrecognized capacity of directly coupling epigenetic and transcriptional regulatory mechanisms to program T_FH_ lineage specification, survival, and functional maturation (Supplementary Figure 8).

## Methods

### Mice

C57BL/6J (B6), B6.SJL, *Ezh2*^fl/fl^, *Arf*^fl/fl^, *Bcl6*^fl/fl^, CD4-Cre transgenic and Rosa26^GFP^ mice were from the Jackson Laboratory. Blimp1-YFP reporter, Tcf1^fl/fl^ and Lef1^fl/fl^ mice were previously described^5,40,67^, hCD2-Cre mice were provided by Paul E. Love (NICHD, NIH)^67^, and *Ink4a*^fl/fl^ mice were provided by Norman Sharpless (NCI). All compound mouse strains used in this work were from in-house breeding at the University of Iowa animal care facility. All mice analyzed were 6–12 weeks of age, and both genders were used without randomization or blinding. All mouse experiments were performed under protocols approved by the Institutional Animal Use and Care Committees of the University of Iowa.

### Flow cytometry, cell sorting, and active caspase detection

Single-cell suspensions were prepared from the spleen, lymph nodes (LNs), and surface or intracellularly stained as described^22,39^. For analysis at 36–60 h and day 4 post-infection, the spleen was first treated with 100 U/ml Collagenase II (Life Technologies) at 37 °C for 15 min to maximize cell recovery. The fluorochrome-conjugated antibodies were as follows: anti-CD4 (RM4–5), anti-CD44 (IM7), anti-CD62L (MEL-14), anti-CD69 (H1.2F3), anti-CD45.2 (104), anti-ICOS (C398.4A), anti-CD25 (PC61.5), Thy1.1 (HIS51), anti-PD-1 (J43), anti-Fas (15A7), anti-GL7 (GL7), anti-IgD (11–26), anti-CD138 (281–2), anti-T-bet (eBio4B10), and rat IgG2a κ isotype control (eBR2a, for intracellular staining of Bcl6) were from eBiosciences; anti-Bcl6 (K112-91) and anti-Ezh2 (11/Ezh2) from BD Biosciences; anti-Irf4 (IRF4.3E4) from BioLegend; anti-Tcf1 (C63D9) and isotype control (Cat. No. 4410S for intracellular staining of Ezh2 and Tcf1) from Cell Signaling Technology; and anti-SLAM (TC15-12F12.2) from BioLegend. For detection of CXCR5, three-step staining protocol was used with unconjugated anti-CXCR5 (2G8; BD Biosciences)^7^. For detection of Bcl6 and Ezh2, surface-stained cells were fixed and permeabilized with the Foxp3/TF Staining Buffer Set (eBiosciences), followed by incubation with corresponding fluorochrome-conjugated antibodies. To preserve Blimp1-YFP detection, the cells were first incubated with 4% formaldehyde at 37 °C for 10 min, chilled on ice for 2 min, and then fixed and permeabilized as above. For detection of pS21-Ezh2 (rabbit polyclonal, Bethyl Laboratories) or pT487-Ezh2 (rabbit polyclonal, abbexa, UK), the surface-stained and fixed cells were first stained with the primary antibody, followed by sequential staining with biotinylated goat anti-rabbit IgG (Cat. No. 111-066-144, Jackson ImmunoResearch Laboratories) and fluorochrome-conjugated streptavidin. For more accurate measurement of the impact of Ezh2 deficiency on Bcl6 expression, Bcl6 expression was specifically analyzed on Ezh2-negative *Ezh2*^–/–^ T_FH_ cells (as in Figs. 3b, 5g, 8e, Supplementary Figures 4d and 5e), excluding about 10% of cells that escaped deletion and retained Ezh2 protein. Active Caspsase-3/7 was detected using the Vybrant FAM caspase-3/7 assay kit (Invitrogen/Life Technologies) as described^68^. Data were collected on an LSRII with Violet and a FACSVerse (BD Biosciences) and were analyzed with FlowJo software V10 (TreeStar). Because Ezh2 deficiency affected CXCR5 expression, for analysis of T_FH_ cells, we first determined the T_FH_ gate based on CXCR5 and SLAM combination in WT cells by following the contour lines, and then applies the same gate to other experimental conditions in each set of experiments. This practice would allow more accurate assessment of the impact on T_FH_ cells by manipulating Ezh2 or downstream target genes. The surface-stained cells were sort-purified on Becton Dickinson Aria II or Aria Fusion at the Flow Cytometry Core Facility of the University of Iowa. The gating strategies for data analysis and cell sorting are illustrated in Supplementary Figure 9.

### Adoptive transfer and viral infection

For direct viral infection, WT, *Ezh2*^–/–^, and Tcf1^–/–^Lef1^–/–^ mice were intraperitoneally (*i.p*.) infected with 2.5 × 10^5^ plaque-forming units (PFU) vaccinia virus (VacV). For adoptive transfer, naïve Smarta CD4^+^ T cells were isolated from the LNs from WT, *Ezh2*^–/–^*, Arf*^–/–^*, Ezh2*^–/–^*Arf*^–/–^, or *Ezh2*^–/–^*Ink4a*^–/–^ Smarta TCR-transgenic mice. For characterization of T_FH_ responses on 4–6 dpi, 5 × 10^4^ Vα2^+^ Smarta CD4^+^ T cells were intravenously (i.v.) injected into CD45.1^+^ B6.SJL recipient mice and i.p. infected with 2 × 10^5^ PFU of LCMV-Arm. To assess cell division, at early T_H_1 and T_FH_ bifurcation (within 72 h after infection), Smarta CD4^+^ T cells were labeled with 10 μM Cell Trace Violet (CTV, Invitrogen/Life Sciences), and 5 × 10^5^ of labeled Vα2^+^ Smarta CD4^+^ cells were transferred followed by i.v. infection with 2 × 10^6^ PFU of LCMV-Arm. To determine basal CTV levels on non-dividing cells in vivo, 2 × 10^6^ CTV-labeled Vα2^+^ Smarta CD4^+^ T cells were i.v. injected into CD45.1^+^ B6.SJL recipient mice and left uninfected.

### Immunization and enzyme-linked immunosorbent assay

The LCMV GP61-82 peptide (CGLNGPDIYKGVYQFKSVEFD) was synthesized and conjugated with KLH to the cysteine by GenScript. GP61-KLH conjugates (40 µg/mouse; 20 µg/rear footpad) were mixed with Addavax (Invivogen) at 1:1 volume ratio, then with polyinosine-polycytidylic acid (4 µg/mouse, Sigma-Aldrich) and used as the immunogen. WT, *Ezh2*^–/–^*, Ezh2*^–/–^*Arf*^–/–^, or retrovirally transduced WT or *Ezh2*^–/–^ Smarta CD4^+^ T cells were adoptively transferred into CD45.1^+^ B6.SJL or CD45.1^+^CD4-Cre^+^*Bcl6*^fl/fl^ (*Bcl6*^–/–^) mice at 2 × 10^5^ cells per recipient. Twenty-four hours later, the recipients were immunized with the immunogen by subcutaneous injection to the rear footpads. On days 5–8 post-immunization, the inguinal LNs were harvested for characterization of T_FH_ cells (usually day 5), and sera were collected for ELISA (day 8).

KLH-specific IgG in the sera was measured by ELISA as previously described^39^. In brief, Nunc MaxiSorp flat-bottom 96-well plate (eBiosciences) was coated with 1 µg/ml Imject mcKLH (Thermo Fisher Scientific) overnight, and then incubated with serially diluted serum samples. The KLH-specific IgG was detected by Horseradish peroxidase (HRP)-conjugated goat-anti-mouse IgG (H+L) secondary antibody (Thermo Fisher Scientific) coupled with TMB substrate (BD Biosciences). The absorbance at 450 nm was read on a Synergy H1M microplate reader (BioTek Instruments).

For antibody response to VacV, the titer of antibody against its D8 envelope protein was determined in the sera of infected mice by ELISA as previously described^69^. In brief, the plate was coated with 1 µg/ml of recombinant D8 protein, incubated with serially diluted serum samples followed by secondary antibody, and then absorbance at 450 nm was detected as above.

### Retroviral transduction and assessment of in vivo rescue effect

*Bcl6* cDNA was cloned into a bicistronic pMSCV-IRES-mCherry retroviral vector (Addgene, Cat. No. #52114). *Ezh2* and *Arf* cDNAs were cloned into another bicistronic pMSCV-IRES-GFP retroviral vector (also known as pMIG, Addgene, Cat. No. #9044), and mutant forms of Ezh2 (S21A and S21D) and Arf (ArfΔ14 and ArfΔ37) were generated and sequences verified. The *Bcl6* promoter region was subcloned upstream of the Thy1.1 cDNA in the dual-reporter self-inactivated retroviral vector with a pQCXIP backbone (Clontech)^40^. The Tcf1 motifs in the *Bcl6* promoter was mutated from CAAAG to AGACA, or CTTTG to TGTCT to create the mutant reporter construct. The retrovirus was packaged in 293T cells as previously described^70^.

WT or *Ezh2*^–/–^ Smarta TCR-transgenic mice were *i.v*. infected with 2 × 10^6^ PFU of LCMV-Arm to prime the Smarta CD4^+^ T cells. One day later, the splenocytes were infected with the retrovirus by spinofection (at 2500 rpm, 37 °C for 90 min), and then cultured overnight in the presence of human IL-2 (100 U/ml) and GP61 peptide (250 nM). The spinofection was repeated the next day, and a total of 2–5 × 10^5^ retrovirally infected Smarta CD4^+^ T cells (containing both infected and uninfected cells) were then adoptively transferred into B6.SJL recipients, followed by either LCMV-Arm infection (2 × 10^5^ PFU) or GP61-KLH immunization. In some experiments, the WT or *Ezh2*^–/–^ Smarta CD4^+^ T cells were enriched with negative selection, and primed in vitro using anti-CD3 and anti-CD28 followed by retroviral transduction. Four days after recipient infection, the mCherry^+^ or GFP^+^ Smarta CD4^+^ T cells were enumerated or phenotypically analyzed.

### Co-immunoprecipitation

The cDNA coding N-terminus FLAG-tagged Tcf1 in the Mig-R1 retroviral vector was described^67^, and the plasmids expressing HA-tagged human Ezh2 and G9a were from Addgene (Cat. No. #24230 and #33024, respectively), and the coding sequence was subcloned into Mig-R1 for expression. The expression plasmids (FLAG-Tcf1 together with either HA-Ezh2 or HA-G9a) were transfected into 293T cells using Lipofectamine 2000 (Life Technologies), and 48 h later, cell lysates were extracted and incubated overnight with 2 µg of anti-FLAG (clone M2, Sigma-Aldrich), followed by 2-h incubation with Dynabeads Protein G (Life Technologies). After proper washing, the immunoprecipitated samples were analyzed by immunoblotting with anti-HA (F7, Santa Cruz Biotechnology). The cell lysates were probed with anti-HA or anti-FLAG to detect input proteins.

In another experiment, *Bcl6*-mCherry and Arf-GFP (WT or mutant forms) were co-transfected into 293T cells, and the cell lysates were immunoprecipitated with anti-Bcl6 (D65C10, Cell Signaling Technologies) and then immunoblotted with anti-p19Arf (5-C3-1, Novus Biologicals), which recognizes amino acids 62–75 of murine p19Arf).

To detect the Ezh2 and Tcf1 interaction in primary T_FH_ cells, CXCR5^+^SLAM^lo^ T_FH_ cells were sorted from mouse splenocytes, on day 8 after LCMV infection. The cell lysate was incubated with 2 µg normal rabbit IgG (Millipore) or 2 µg anti-Tcf1 (C63D9, Cell Signaling Technologies) for 5 h at 4 °C with rocking, followed by a 5-hour incubation with Dynabeads Protein G. After proper washing, the immunoprecipitated samples were immunoblotted with anti-Ezh2 (AC22, Active Motif). Uncropped gel images of all immunoblotting experiments are shown in Supplementary Figure 10.

### Quantitative RT-PCR

CD45.2^+^ T_FH_ cells were sorted from the spleens of recipient mice on 4 dpi with LCMV-Arm. Total RNA was extracted, reverse-transcribed, and target gene transcripts were measured with quantitative PCR as described^67^. The primers used are listed in Supplementary Table 1.

RNA-Seq and data analysis. WT and *Ezh2*^–/–^ mice were infected with VacV, and on 8 *dpi*, CXCR5^+^PD-1^–^ T_FH_ cells were sorted and total RNA was extracted. Two biological replicates were obtained for each genotype, and used for RNA-Seq analysis as previously described^7^. The sequencing quality of RNA-Seq libraries was assessed by FastQC v0.10.1 (http://www.bioinformatics.babraham.ac.uk/projects/fastqc/). RNA-Seq libraries were mapped to mouse genome using Tophat (v2.1.0)^71^, and the mapped reads were then processed by Cuffdiff (v2.2.1)^72^ to estimate expression levels of all genes and identify differentially expressed genes. The expression level of a gene is expressed as a gene-level Fragments Per Kilobase of transcripts per Million mapped reads (FPKM) value. Upregulated or downregulated genes in *Ezh2*^−/−^ T_FH_ cells were identified by requiring ≥ 2-fold expression changes and FDR < 0.05, as well as FPKM ≥ 1 in *Ezh2*^−/−^ T_FH_ cells for upregulated genes, or FPKM ≥ 1 in WT T_FH_ cells for downregulated genes. UCSC genes from the iGenome mouse mm9 assembly (https://support.illumina.com/sequencing/sequencing_software/igenome.html) were used for gene annotation. The RNA-Seq data are deposited at the GEO (accession number 103387).

### Gene set enrichment analysis

GSEA was performed with GSEA software from the Broad Institute^73^, and used to determine enrichment of gene sets of interest, in *Ezh2*^−/−^ or WT T_FH_ cells. The gene set of “Tcf1-activated genes in T_FH_ cells” contains 513 genes that are downregulated by ≥1.5 fold in CD4-Cre^+^Tcf1^fl/fl^ T_FH_ cells (GSE65693)^9^; the gene set of “Tcf1/Lef1-activated genes in GC T_FH_ cells” contains 306 genes that are downregulated by ≥1.5 fold in hCD2-Cre^+^Tcf1^fl/fl^Lef1^fl/fl^ GC-T_FH_ cells (GSE66781)^7^; and the gene set of “T_FH_-enrich genes” contains 491 genes that are expressed at ≥2 fold higher in T_FH_ than T_H_1 cells (GSE21380)^7^. Ezh2/Tcf1 co-bound genes in T_FH_ cells were identified in this work.

### Chromatin immunoprecipitation

For detection of histone marks, WT or *Ezh2*^–/–^ Smarta CD4^+^ T cells were adoptively transferred into B6.SJL recipients followed by LCMV-Arm infection. On 4 dpi, CXCR5^+^SLAM^lo^ T_FH_ cells were sort-purified, cross-linked with 1% formaldehyde in media for 5 min, processed using truChIP Chromatin Shearing Reagent Kit (Covaris), and sonicated for 5 min on a Covaris S2 ultrasonicator. The sheared chromatin was immunoprecipitated with anti-H3K27me3 (Millipore, 17–622) or anti-H3K27ac (Abcam, ab4729) and washed as previously described^74^. The immunoprecipitated DNA segments were used for quantification by PCR. For calculation of enrichment of histone marks, the signal at the genomic region of interest in each ChIP sample were first normalized to input DNA, and then normalized to a negative control region that is devoid of histone modification, as defined previously^74^. The histone mark signal in WT cells was set as 1, and that in *Ezh2*^–/–^ cells were calculated accordingly.

For detection of Ezh2 binding, WT or Tcf1^–/–^Lef1^–/–^ mice were infected with LCMV-Arm, and on 6 dpi, CXCR5^+^SLAM^lo^ T_FH_ cells were sort-purified and processed as above (except that the cells were crosslinked with formaldehyde for 10 min instead). The sheared chromatin was immunoprecipitated with 4 µg of anti-Ezh2 (Cat. No. ab3748, Abcam) or normal rabbit IgG (Cat. No. CS200581, EMD Millipore) and washed as described^39^. To calculate enriched Ezh2 binding, the signal at the genomic region of interest in each Ezh2 ChIP sample were first normalized to that in IgG ChIP, and the relative enrichment by anti-Ezh2 was then normalized to that at the *Hprt* promoter. The primers used are listed in Supplementary Table 1.

### ChIP-Seq and data processing

WT mice were infected with VacV, and on 8 dpi, CXCR5^+^PD-1^lo^ T_FH_ cells were sort-purified, processed and subject to ChIP analysis with anti-Ezh2, anti-Tcf1 (homemade as previously described^75^), anti-H3K27me3 or anti-H3K27ac as above. For negative controls, Ezh2- and Tcf1-deficient naïve CD4^+^ T cells were enriched by negative selection from the splenocytes of Ezh2^–/–^ and CD4-Cre^+^Tcf1^fl/fl^ mice, respectively. DNA segments from ChIP or input DNA were end-repaired and ligated to indexed Illumina adaptors followed by low-cycle PCR. The resulting libraries were sequenced with the Illumina HiSeq-2000 platform. The ChIP-Seq data are under GEO accession number GSE103387.

The sequencing quality of ChIP-Seq libraries was assessed by FastQC. Bowtie2 v2.2.6^76^ was used to align the sequencing reads to the mm9 mouse genome. UCSC genes from the iGenome mouse mm9 assembly were used for gene annotation. Mapped reads were processed with SICER (v1.1)^38^ for peak calling with the setting of FDR < 10^−4^. We identified 6,130 Ezh2 binding peaks in T_FH_ cells with Ezh2-deficient CD4^+^ T cells as a negative control, and 11,561 Tcf1 binding peaks in T_FH_ cells with Tcf1-deficient CD4^+^ T cells as a negative control. Histone mark enriched regions were identified using SICER (V1.1) at FDR < 10^−4^ with input DNA as a negative control.

To generate Ezh2 and Tcf1 colocalization heatmaps, Ezh2 and Tcf1 peaks within +/–10 kb of gene TSSs are divided into three groups: 2,219 Ezh2^+^Tcf1^+^ peaks (group 1), 2,615 Ezh2^+^Tcf1^–^ peaks (group 2), and 3,692 Ezh2^–^Tcf1^+^ peaks (group 3). The Ezh2 peak summits in groups 1 and 2, and Tcf1 summits in group 3, are used as the center of alignment. The island-filtered reads of Ezh2 or Tcf1 ChIP-Seq, expressed as log(RPKM + 1) values, were shown in the heatmaps. The reads of H3K27me3 and H3K27ac associated with Ezh2 and/or Tcf1 peaks were processed similarly to generate respective heatmaps. Within each group, the peaks were ordered according to H3K27me3 level from low to high, and then ordered according to H3K27ac level from high to low in case of H3K27me3 degeneracy. For motif analysis of the 2,219 Ezh2^+^Tcf1^+^ peaks, the sequences of ±200 bps flanking the peak summits, as identified by SICER, were used in MEME-ChIP for de novo motif discovery^77^.

### Statistical analysis

For comparison between two experimental groups, Student’s *t*-test with two-tailed distribution was used. For multiple group comparisons, one way ANOVA was used to first determine whether any of the differences among the means are statistically significant, followed by (1) unpaired Student’s *t*-test to determine the statistical significance for a specific pair, or (2) post hoc tests using Bonferroni’s test to more stringently determine the statistical significance of differences between all possible pairs.

### Reporting summary

Further information on experimental design is available in the Nature Research Reporting Summary linked to this article.

## Supplementary information

Supplementary Information

Peer Review File

Reporting Summary

## Data Availability

The RNA-Seq data on WT and *Ezh2*^–/–^ T_FH_ cells, along with Tcf1, Ezh2, H3K27ac and H3K27me3 ChIP-Seq in WT T_FH_ cells are deposited at the Gene Expression Omnibus under accession number GSE103387.
