# Supplementary material for: Ezh2 programs TFH differentiation by integrating phosphorylation-dependent activation of Bcl6 and polycomb-dependent repression of p19Arf
Source: Nat Commun. 2018 Dec 21;9:5452. doi: 10.1038/s41467-018-07853-z (PMC6303346; doi:10.1038/s41467-018-07853-z)
Supplement: Supplementary file 1 — Supplementary Information [file 41467_2018_7853_MOESM1_ESM.pdf]

Ezh2 programs T_{FH} differentiation by integrating phosphorylation-dependent activation of Bcl6 and polycomb-dependent repression of p19Arf

Li et al.

Supplementary information

Li et al. Supplementary Figure 1

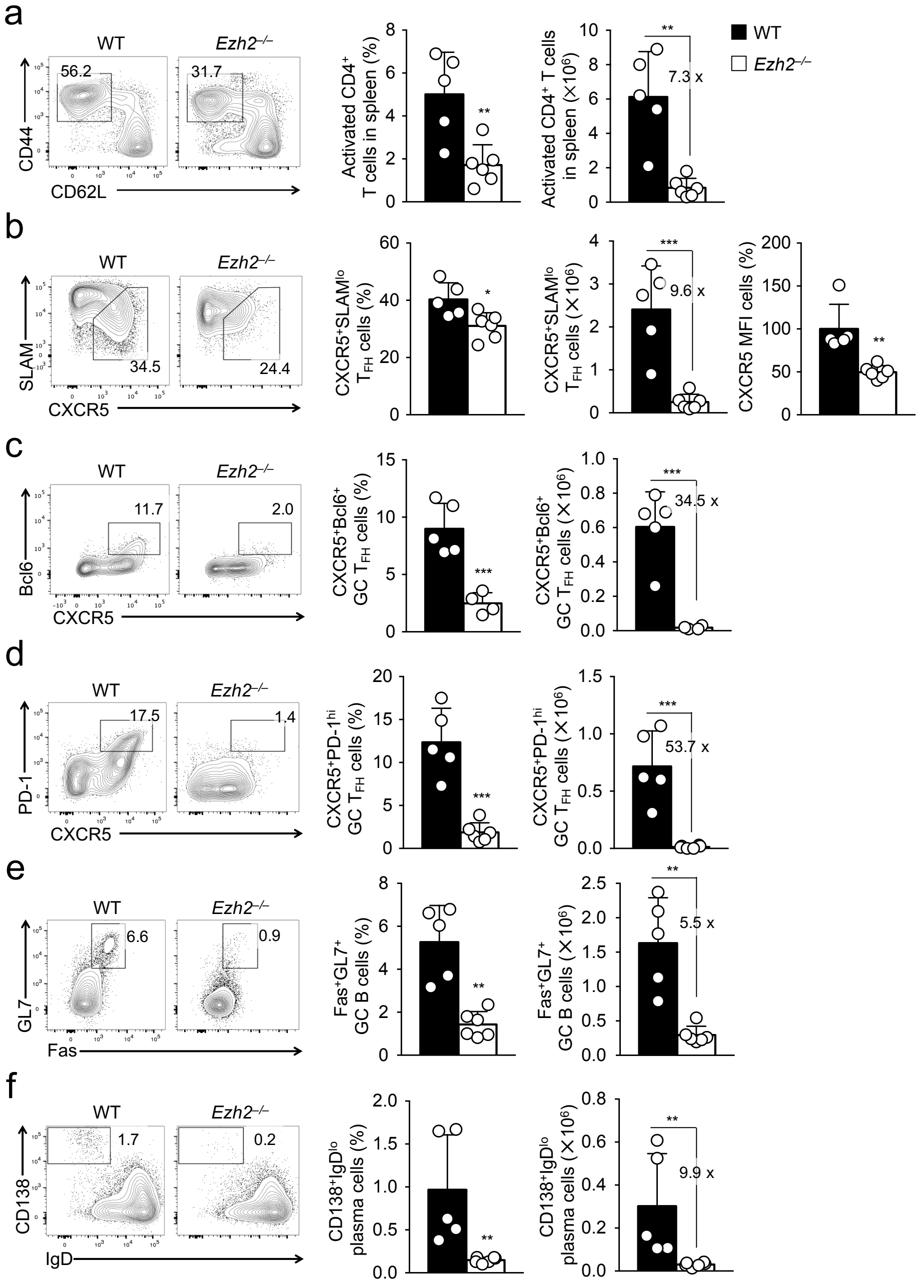

Supplementary Figure 1. Ezh2 is required for T_{FH} and B cell responses to acute viral infection.

WT and *Ezh2*$^{-/-}$ mice were *i.p.* infected with LCMV-Arm. On 8 *dpi*, CD44^{hi}CD62L^{-} activated CD4^{+} T cells were detected in the spleen (**a**), and further analyzed for CXCR5^{+}SLAM^{lo} T_{FH} cells (**b**), CXCR5^{+}Bcl6^{+} (**c**) and CXCR5^{+}PD-1^{hi} GC-T_{FH} cells (**d**). Splenic B220^{+}CD19^{+} cells were analyzed for Fas^{+}GL7^{+} GC B cells (**e**) and CD138^{+}IgD^{lo} plasma cells (**f**). Contour plots are representative from two experiments, and cumulative data on frequency and numbers of each subset are means ± s.d. and each dot represents one mouse analyzed. Also shown in **b** is the relative CXCR5 gMFI. *, p<0.05; ** p <0.01; ***, p<0.001 by unpaired, two-tailed Student's *t*-test.

Li et al. Supplementary Figure 2

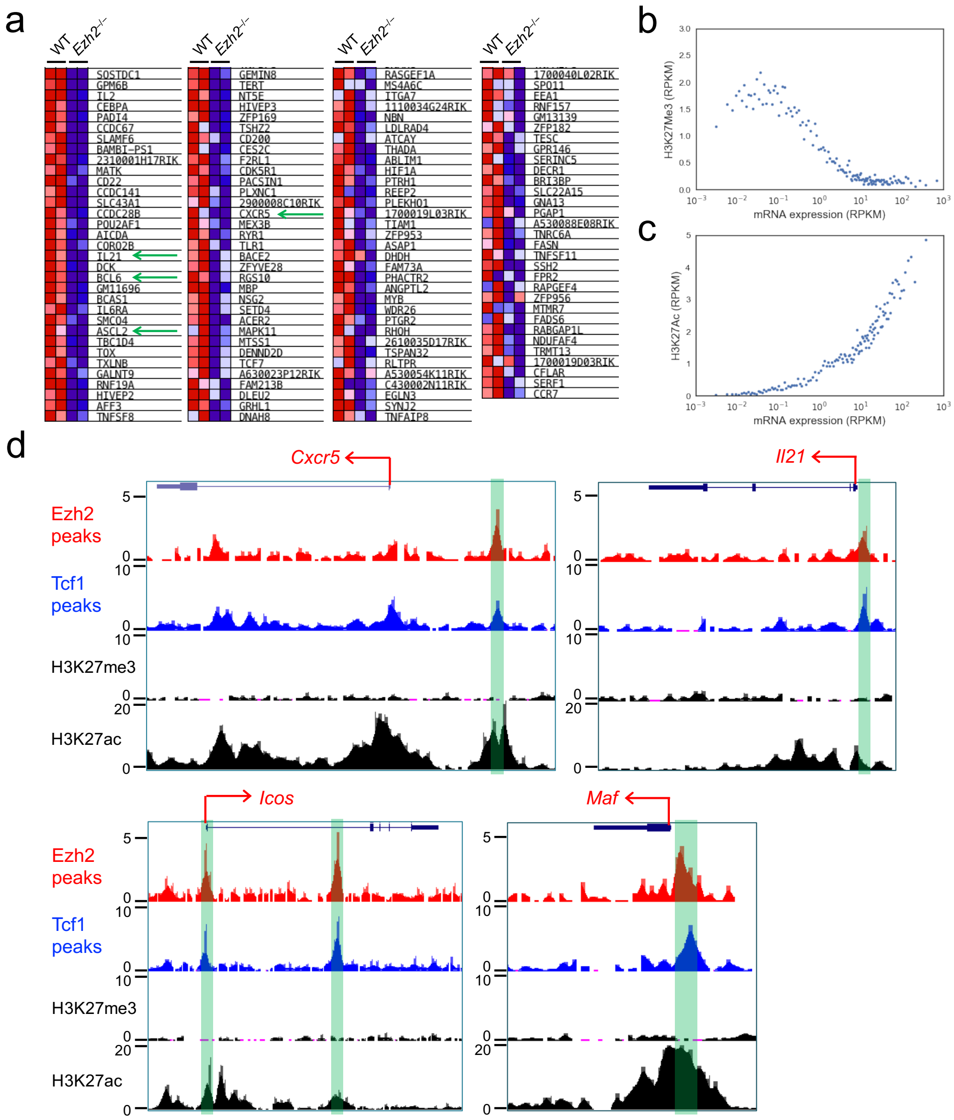

Supplementary Figure 2. Ezh2 is predominantly associated with activation of T_{FH} program.

(**a**) Heatmap of genes in the T_{FH}-associated gene set that was negatively enriched in *Ezh2*$^{-/-}$ T_{FH} cells, as determined by GSEA in **Figure 2d**.
(**b–c**) Gene expression in WT T_{FH} cells was negatively correlated with H3K27me3 but positively correlated with H3K27ac levels. WT C57BL/6 mice were infected with VacV, and on 8 *dpi*, $CXCR5^{+}PD\text{-}1^{lo}$ T_{FH} cells were sorted and subjected to RNA-Seq analysis and ChIP-Seq of H3K27me3 and H3K27ac. A total of 23,680 annotated mouse genes are divided into 200 equally populated groups based on the gene expression level. For measurement of histone mark levels associated with a gene, the island-filtered reads of H3K27me3 or H3K27ac were counted from –5 kb upstream of transcription start to end site of the gene, and then normalized to the gene length and the library size. The average normalized levels of H3K27me3 (**b**) or H3K27ac (**c**) associated with genes in each group were then plotted against the average gene expression in the group.
(**d**) ChIP-Seq tracks of Ezh2, Tcf1, H3K27me3 and H3K27ac at the *Cxcr5, Il21, Icos* and *Maf* gene loci are displayed on the UCSC genome browser, with gene structure and transcription orientation marked on top. Vertical green bars denote Ezh2 and Tcf1 co-occupied sites.

Li et al. Supplementary Figure 3

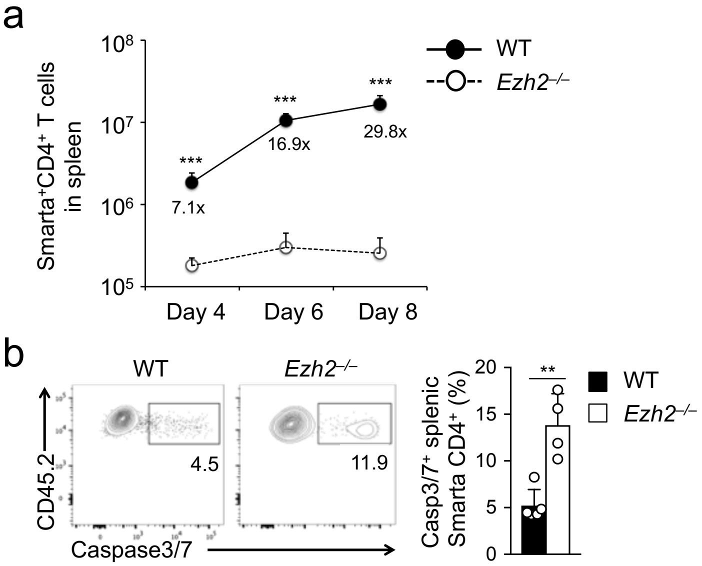

Supplementary Figure 3. Loss of Ezh2 impairs Smarta CD4$^+$ T cell expansion and survival.

Naïve *Ezh2*$^{-/-}$ or WT Smarta CD4$^+$ T cells were adoptively transferred into CD45.1$^+$ congenic recipients followed by LCMV-Arm infection. On 4, 6, and 8 *dpi*, the numbers of Smarta CD4$^+$ T cells were determined in the spleen of recipients (**a**). Caspase-3/7 activation was detected in Smarta CD4$^+$ T cells on 4 *dpi* (**b**). Cumulative data are means ± s.d. (n = 6 from 2 experiments). ** p <0.01; ***, p<0.001 by Student's *t*-test.

Li et al. Supplementary Figure 4

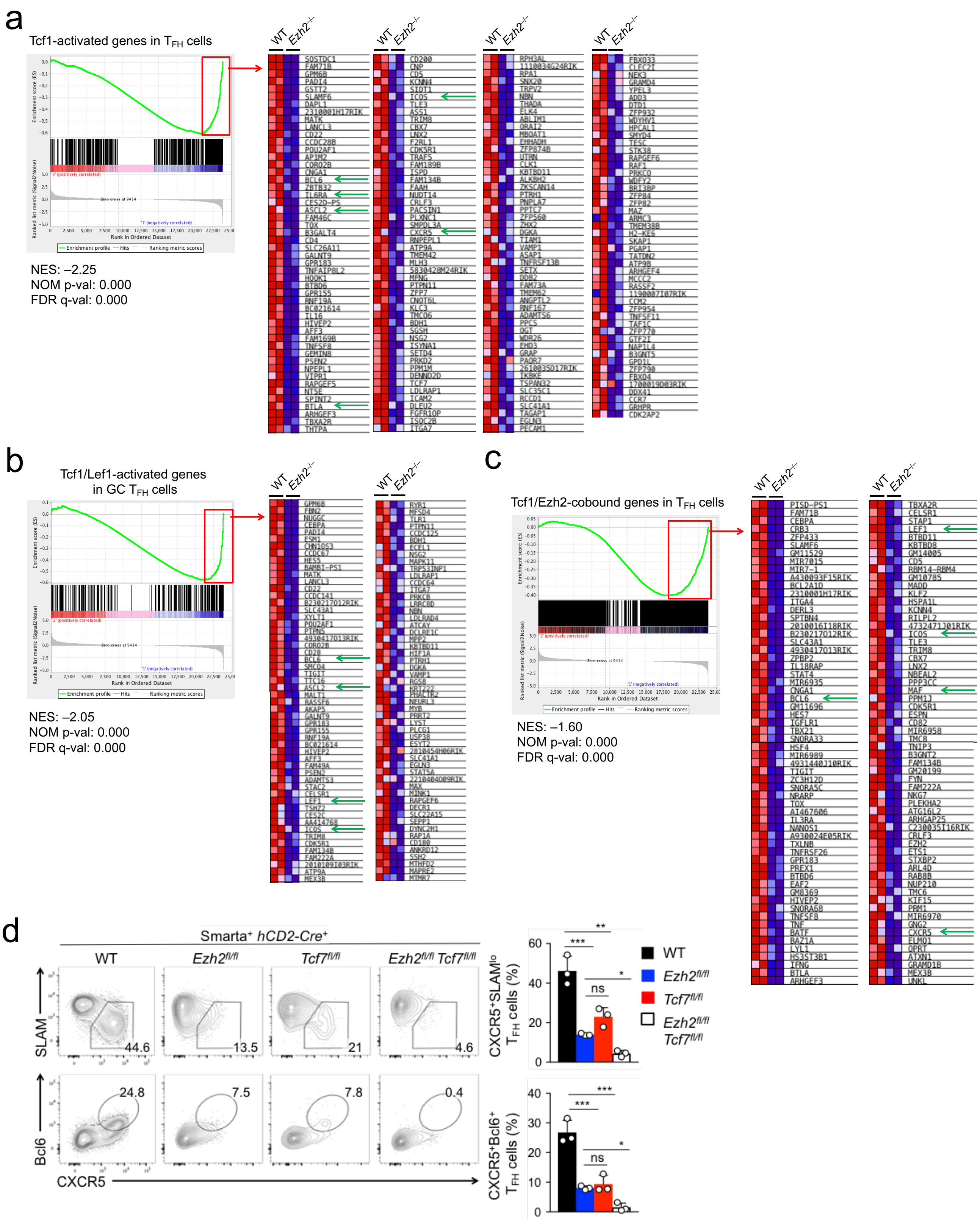

Supplementary Figure 4. Tcf1 and Ezh2 share common target genes in the T_{FH} program.

(**a–c**) GSEA analysis of Tcf1/Lef1-dependent genes in Ezh2-deficient T_{FH} cells. NES, normalized enrichment scores; NOM p-val, nominal p values; FDR q-val, false discovery rate q values.

(**a**) The gene set of "Tcf1-activated genes in T_{FH} cells" was from *Xu et al. Nat. Immunol. 2015 (Ref. 9)*, where *CD4-Cre*$^{+}$Tcf1$^{fl/fl}$ and control mice were infected with LCMV-Arm, and T_{FH} cells were sorted and analyzed on microarray. This analysis identified 513 genes that were downregulated by ≥1.5 fold in CD4-Cre^{+}Tcf1$^{fl/fl}$ T_{FH} cells, and by GSEA, this gene set was negatively enriched in Ezh2$^{-/-}$ T_{FH} cells, with 196 genes (~39%) at the leading edge of enrichment curve showing diminished expression.

(**b**) The gene set of "Tcf1/Lef1-activated genes in GC-T_{FH} cells" was from *Choi et al. Nat. Immunol. 2015 (Ref. 7)*, where human CD2-Cre^{+}Tcf1$^{fl/fl}$Lef1$^{fl/fl}$ and littermate controls were infected with VacV, and CXCR5^{+}PD-1^{+} GC-T_{FH} cells were sorted and analyzed by RNA-Seq. This analysis identified 306 genes that were downregulated by ≥1.5 fold in *hCD2-Cre*$^{+}$Tcf1$^{fl/fl}$Lef1$^{fl/fl}$ GC-T_{FH} cells, and by GSEA, this gene set was negatively enriched in Ezh2$^{-/-}$ T_{FH} cells, with 108 genes (~35%) at the leading edge of enrichment curve showing diminished expression.

(**c**) The gene set of "Ezh2/Tcf1 co-bound genes" was from this study (derived from group 1 in **Fig. 2e**). Ezh2/Tcf1 co-bound gene set consists of genes that contain Ezh2/Tcf1 co-occupied site(s) within +/–10 kb region flanking TSSs. By GSEA, this gene set was negatively enriched in Ezh2$^{-/-}$ T_{FH} cells, with 1,027 out of a total of 2,975 genes (~34.5%) at the leading edge of enrichment curve showing diminished expression.

Shown on left panels are the enrichment plots, and the negatively enriched genes, highlighted with red rectangles, are displayed in the heatmap on right panels (all genes for **a** and **b**, and top 120 genes for **c**). Green arrows highlight key genes with known biological functions in T_{FH} cells.

(**d**) Combined deletion of Ezh2 and Tcf1 almost completely abolishes T_{FH} differentiation. *Ezh2*$^{fl/fl}$ and *Tcf7*$^{fl/fl}$ mice were crossed to *hCD2-Cre* transgene to ablate targeted genes specifically in mature T cells. CD45.2^{+} Smarta CD4^{+} T cells from the LNs of WT, *hCD2-Cre*$^{+}$*Ezh2*$^{fl/fl}$, *hCD2-Cre*$^{+}$*Tcf7*$^{fl/fl}$, *hCD2-Cre*$^{+}$*Ezh2*$^{fl/fl}$*Tcf7*$^{fl/fl}$ Smarta-Tg mice were adoptively transferred into congenic mice followed by infection with LCMV-Arm. On 4 *dpi*, activated CD45.2^{+} Smarta CD4^{+} T cells were detected in the recipient spleens. Because *hCD2-Cre*-mediated target gene deletion is not complete (ranging at 70-90%), we performed intracellular staining of Ezh2 or Tcf1 and specifically analyzed Ezh2-negative subset in *hCD2-Cre*$^{+}$*Ezh2*$^{fl/fl}$ or *hCD2-Cre*$^{+}$*Ezh2*$^{fl/fl}$*Tcf7*$^{fl/fl}$, and Tcf1-negative subset in *hCD2-Cre*$^{+}$*Tcf7*$^{fl/fl}$ Smarta CD4^{+} T cells. These subsets were further analyzed for CXCR5^{+}SLAM^{lo} (top) or CXCR5^{+}Bcl6^{+} T_{FH} cells (bottom panels). Cumulative data on the frequency of each population are means ± s.d. (n = 3). *, $p<0.05$, ** $p<0.01$; ***, $p<0.001$ by Student's *t*-test for indicated pairwise comparison.

Li et al. Supplementary Figure 5

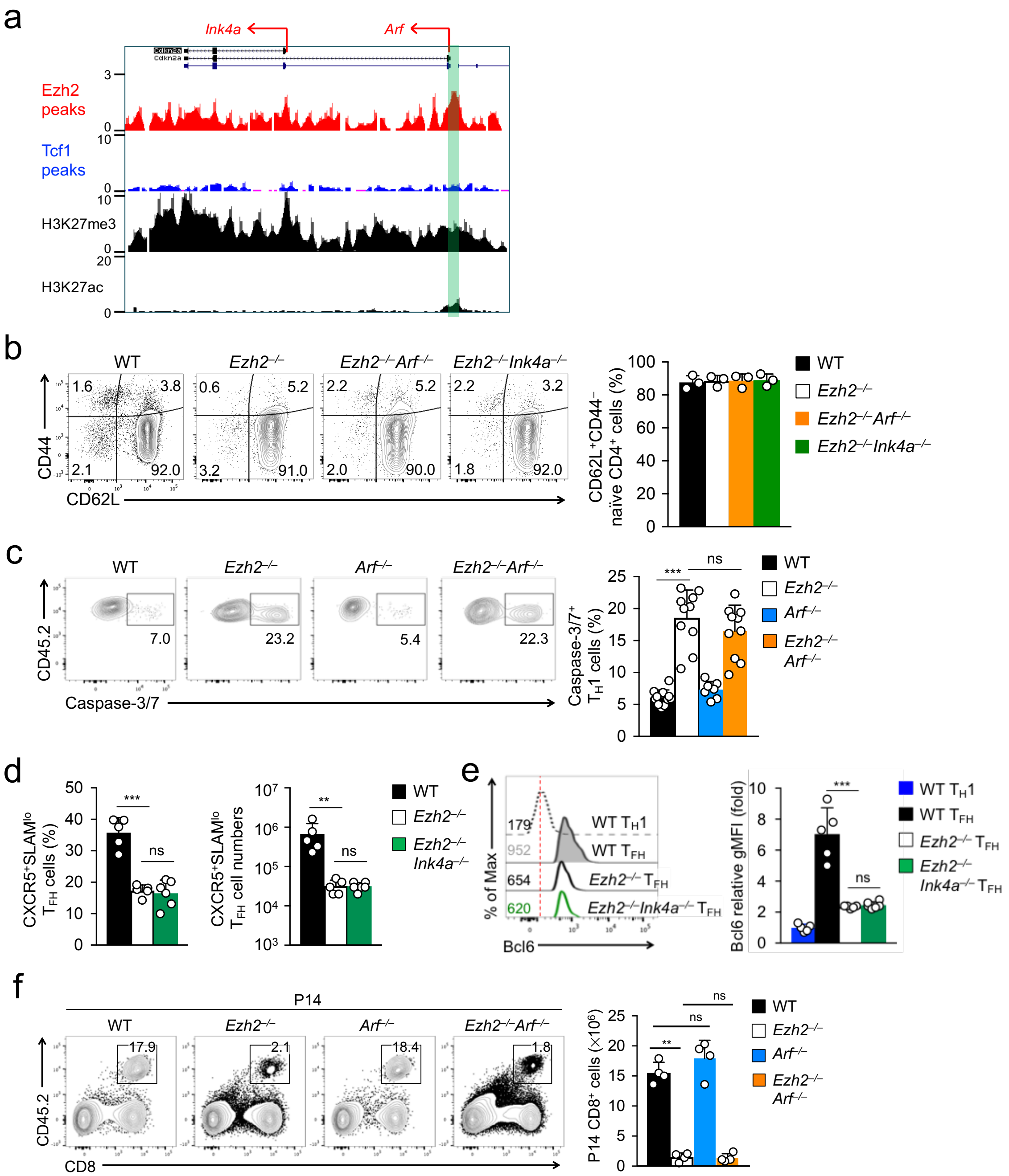

Supplementary Figure 5. Ezh2-dependent T_{FH} differentiation does not require p16Ink4a repression.

(**a**) ChIP-Seq tracks of Ezh2, Tcf1, H3K27me3 and H3K27ac at the *Cdkn2a* gene locus, as displayed on the UCSC genome browser. Also marked are the TSSs of *Arf* and *Ink4a*.

(**b**) Loss of Ezh2 alone or in combination with p19Arf or p16Ink4a does not cause aberrant T cell activation. Smarta $CD4^+$ T cells from the LNs of WT, *Ezh2*$^{-/-}$, *Ezh2*$^{-/-}$*Arf*$^{-/-}$, *Ezh2*$^{-/-}$*Ink4a*$^{-/-}$ Smarta-Tg mice were analyzed for CD44 and CD62L expression, and the frequency of $CD44^{lo}CD62L^+$ naïve $CD4^+$ T cells is summarized in a bar graph (n = 3 from 2 experiments).

(**c**) Ablation of p19Arf does not rescue T_H1 survival defects caused by Ezh2 deficiency. $CD45.2^+$ Smarta $CD4^+$ T cells from WT, *Ezh2*$^{-/-}$, *Arf*$^{-/-}$, or *Ezh2*$^{-/-}$*Arf*$^{-/-}$ Smarta-Tg mice were adoptively transferred into congenic mice followed by infection with LCMV-Arm. On 4 *dpi*, $CXCR5^-SLAM^{hi}$ T_H1 cells were analyzed for caspase-3/7 activation.

(**d–e**) Ablation of p16Ink4a does not rescue T_{FH} defects caused by Ezh2 deficiency. $CD45.2^+$ Smarta $CD4^+$ T cells from the LNs of WT, *Ezh2*$^{-/-}$, *Ezh2*$^{-/-}$*Ink4a*$^{-/-}$ Smarta-Tg mice were adoptively transferred into congenic mice followed by infection with LCMV-Arm. On 4 *dpi*, $CXCR5^+SLAM^{lo}$ T_{FH} cells were detected in the recipient spleens (**d**), and further analyzed for Bcl6 expression (**e**). In **e**, Bcl6 expression is also determined in $CXCR5^-SLAM^{hi}$ T_H1 cells for direct comparison with that in T_{FH} cells, values denote gMFI, and dotted red line marks histogram peak in Bcl6 staining in WT T_H1 cells.

(**f**) Ezh2 deficiency compromises effector $CD8^+$ T cell expansion, which is not rectified by compound deletion of p19Arf. $CD45.2^+$ P14 $CD8^+$ T cells from WT, *Ezh2*$^{-/-}$, *Arf*$^{-/-}$, or *Ezh2*$^{-/-}$*Arf*$^{-/-}$ P14-Tg mice were adoptively transferred into congenic mice (2×10^4 cells for each recipients) followed by infection with LCMV-Arm. On 6 *dpi*, $CD45.2^+CD8^+$ T cells were detected and enumerated.

Data in **c–f** are means ± s.d. from 2 independent experiments ($n \geq 4$). ns, not statistically significant, ** $p <0.01$; ***, $p<0.001$ by Student's *t*-test for indicated pairwise comparison, coupled with one-way ANOVA for multi-group comparisons.

Li et al. Supplementary Figure 6

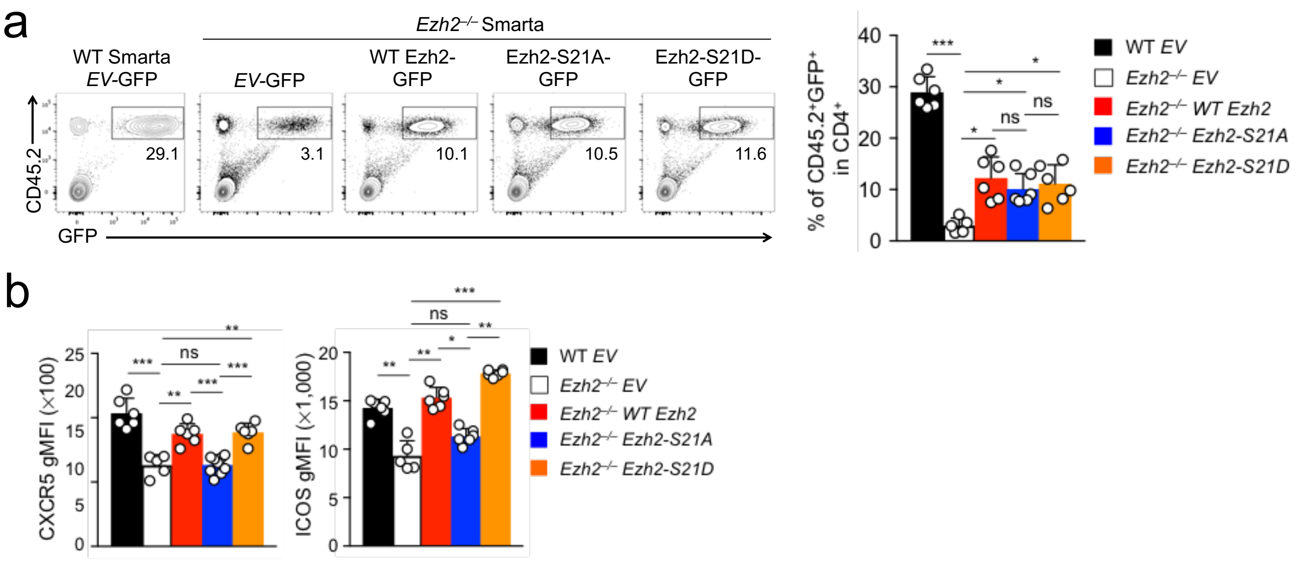

Supplementary Figure 6. Phosphorylation-resistant Ezh2 is ineffective in rectifying T_{FH} differentiation defects in Ezh2-deficient $CD4^+$ T cells.

Ezh2$^{-/-}$ Smarta $CD4^+$ T cells were primed and transduced with *EV*-GFP retrovirus or that expressing WT Ezh2, phosphorylation-resistant Ezh2-S21A, or phosphomimetic Ezh2-S21D, followed by adoptive transfer and LCMV-Arm infection. WT Smarta cells infected with *EV*-GFP were used as a control. On 4 *dpi* (equivalent to day 7 after initial $CD4^+$ T cell priming), the frequency of $GFP^+CD45.2^+$ cells was determined in all $CD4^+$ T cells in recipient spleens (**a**). $CXCR5^+SLAM^{lo}$ T_{FH} cells were identified among $GFP^+CD45.2^+CD4^+$ T cells, and analyzed for CXCR5 and ICOS protein expression (geometric MFI, **b**). Cumulative data are means ± s.d. from ≥ 2 independent experiments (n ≥ 3). ns, not statistically significant; *, p<0.05; ** p <0.01; ***, p<0.001 by Student's *t*-test indicated pairwise comparison, coupled with one-way ANOVA for multi-group comparison.

Li et al. Supplementary Figure 7

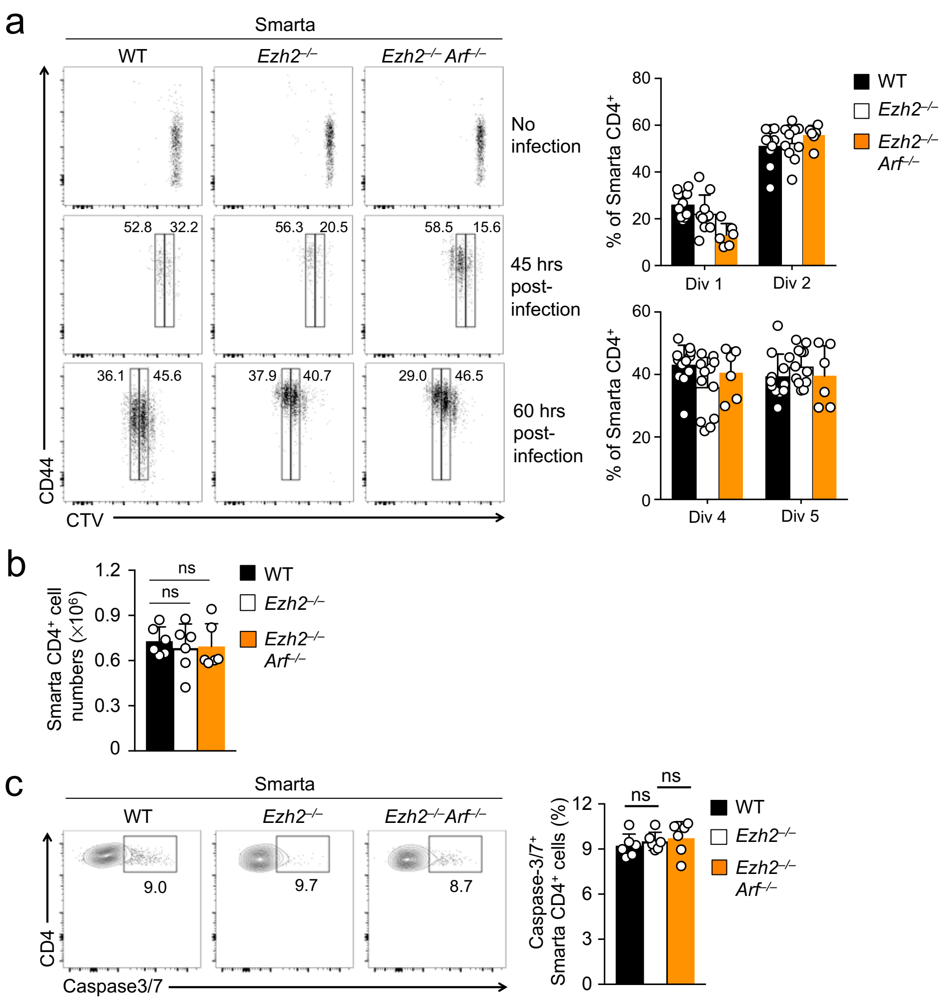

Supplementary Figure 7. Loss of Ezh2 does not affect CD4$^+$ T cell activation and early division.

(**a**) Detection of early division of Smarta CD4$^+$ T cells upon activation. CD45.2$^+$ Smarta CD4$^+$ T cells from the LNs of WT, *Ezh2*$^{-/-}$, or *Ezh2*$^{-/-}$*Arf*$^{-/-}$ Smarta-Tg mice were labeled with Cell Trace Violet (CTV) and adoptively transferred into CD45.1$^+$ congenic recipient mice. One cohort of recipients was left uninfected for detection of CTV levels in non-dividing cells (top panel), and the rest was *i.v.* infected with LCMV-Arm. At 45 and 60 hrs post-infection (middle and bottom panels, respectively), CD44 expression and dilution of CTV in CD45.2$^+$ Smarta CD4$^+$ T cells were detected in the spleen. Representative dot plots are from ≥ 2 experiments (n ≥ 5). For clarity, only 1st and 2nd divisions are marked for 45 hrs, and 4th and 5th divisions marked for 60 hrs post-infection. Values denote percentage in each division. Cumulative data are summarized as means ± s.d.

(**b**) Accumulation of activated Smarta CD4$^+$ T cells is not affected by loss of Ezh2 during the early activation stage. At 60 hrs post-infection, CD45.2$^+$ Smarta CD4$^+$ T cells were detected in the recipient spleens and enumerated.

(**c**) Detection of Caspase-3/7 activation in Smarta CD4$^+$ T cells at 60 hrs post-infection. Data are means ± s.d. from 2 independent experiments (n ≥ 5). ns, not statistically significant by Student's *t*-test for indicated pairwise comparison.

Li et al. Supplementary Figure 8

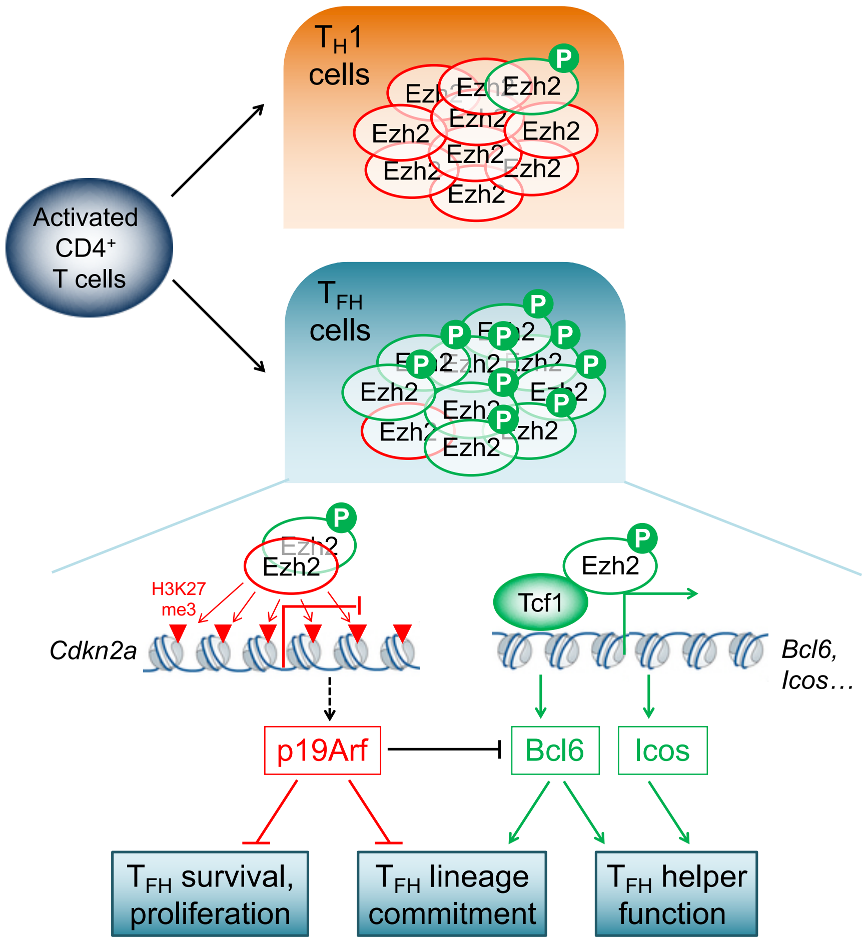

Supplementary Figure 8. Proposed model for Ezh2 in integrating transcriptional activation and epigenetic silencing mechanisms to promote T_{FH} differentiation.

Upon activation of $CD4^+$ T cells in response to viral infections, Ezh2 is rapidly upregulated and phosphorylated at the Ser21 residue even before the first division. At the 4th and 5th divisions within 60 hrs of activation when the T_H1 and T_{FH} lineage bifurcation occurs, Ser21-phosphorylated Ezh2 is predominantly associated with the early T_{FH} cells. Ezh2 remains in Ser21-phosphorylated form in committed T_{FH} cells during further differentiation. Ezh2 regulates T_{FH} lineage specification and continued differentiation through two distinct mechanisms. The first is HMT-dependent repression of p19Arf, one protein product encoded by the *Cdkn2a* gene, regardless of Ezh2 phosphorylated or unphosphorylated status. This function has double impact, 1) a conventional effect of promoting cell survival and/or proliferation and 2) a T_{FH}-specific effect of preventing inhibition of Bcl6. The second mechanism requires the Ser21-phosphorylated form of Ezh2 to promote Bcl6 induction and activation of the T_{FH} transcriptional program. Therefore, Ezh2 has a unique capacity of coupling epigenetic and transcriptional regulation to program T_{FH} differentiation.

Li et al. Supplementary Figure 9

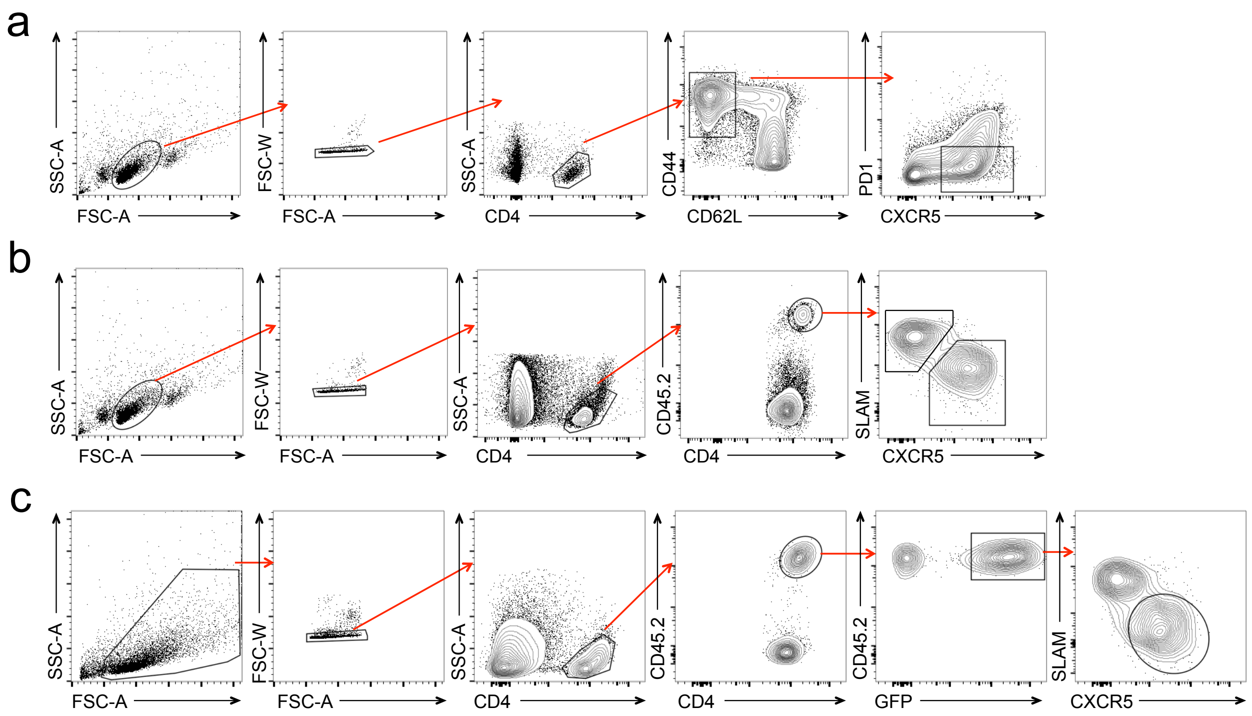

Supplementary Figure 9. Gating strategies for flow cytometry data analysis and cell sorting.

(**a**) Analysis of polyclonal T_{FH} cell responses to acute viral infection, including phenotypic characterization (Figures 1 and 7), RNA-Seq and ChIP-Seq analyses (Figures 2 and 4), and immunoblotting analysis (Figures 4 and 7).

(**b**) Analysis of monoclonal Smarta T_{FH} cell responses to LCMV-Arm infection, including phenotypic characterization (Figures 3, 5, 6 and 8), quantitative RT-PCR and ChIP-PCR analyses (Figures 3, 5 and 7).

(**c**) Analysis of T_{FH} cell responses after retroviral transduction, including phenotypic characterization (Figures 3, 4, 6 and 7), quantitative RT-PCR and ChIP-PCR (Figure 7).

Li et al. Supplementary Figure 10

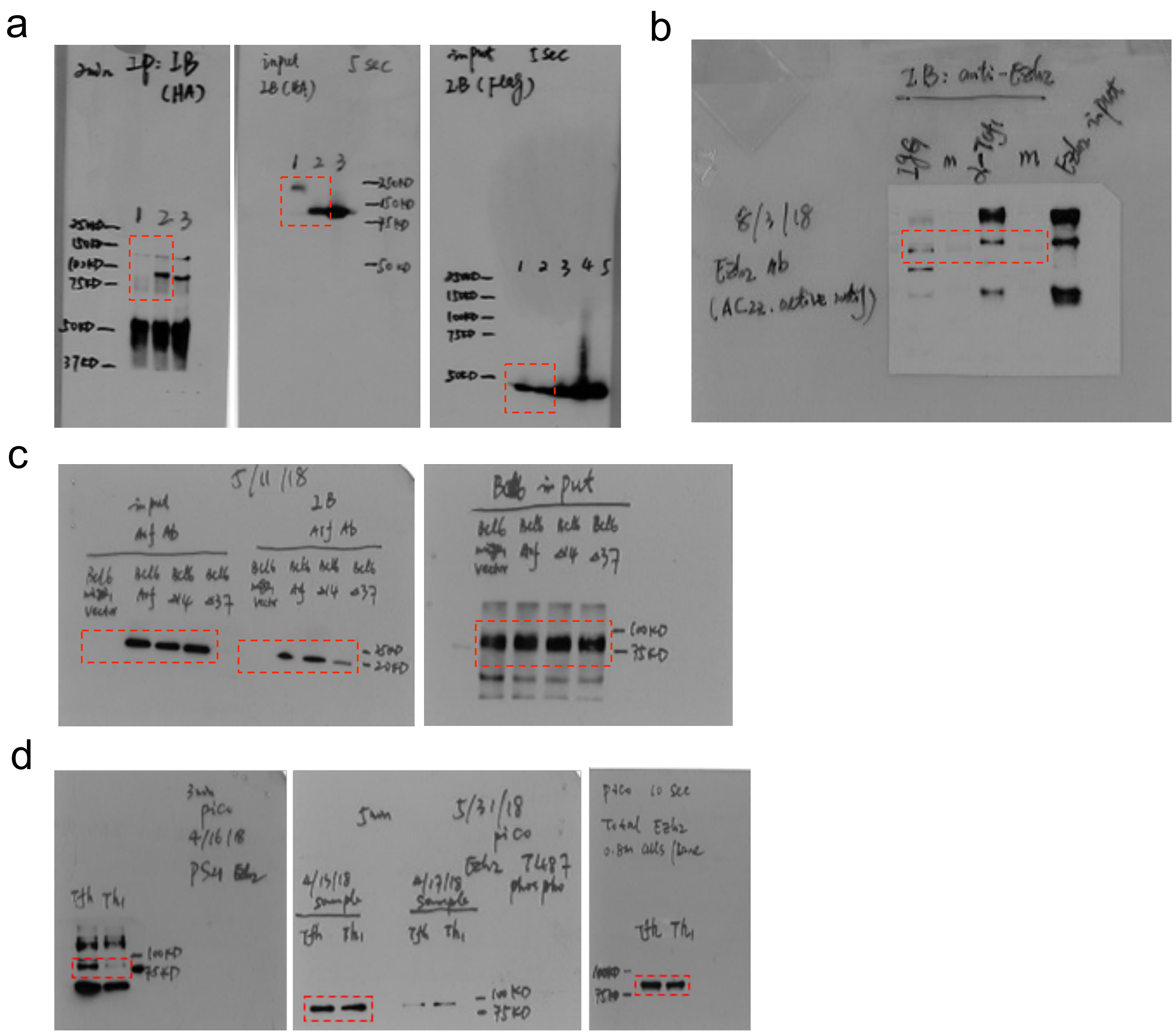

Supplementary Figure 10. Source data of uncropped images in immunoblotitng experiments.

(**a**) Uncropped images for Figure 4d, 3 panels.

(**b**) Uncropped images for Figure 4e, 1 panel.

(**c**) Uncropped images for Figure 6b, 3 panels.

(**d**) Uncropped images for Figure 7a, 3 panels.

Red dotted rectangles denote areas that were extracted for presentation in main figures.

Li et al. Supplementary Table 1

Gene symbol	5' primer	3' primer
**For gene expression analysis**		
Hprt1	5'-GCGTCGTGATTAGCGATGATG	5'-CTCGAGCAAGTCTTTCAGTCC
Bcl6	5'-CCTGAGGGAAGGCAATATCA	5'-CGGCTGTTCAGGAACTCTTC
Arf	5'-TGGTCACTGTGAGGATTCAGC	5'-TTGCCCATCATCATCACCTGG
Ink4a	5'-AAAGCGAACTCGAGGAGAGC	5'-TCATCATCACCTGAATCGGGG
**For Ezh2 occupancy in genomic locations**		
Hprt1	5'-CCTCTGCCTCCTAAATGCTG	5'-TGTCGTCTCCCAGAGGATTC
Bcl6 TSS	5'-TTCGGTGACATAAGAGGGAGA	5'-AGCTGGAAGGAGCTGTGGTA
Arf TSS	5'-TAGCAGTAGCTGCGCCCTTT	5'-CCTCGCCGATCTTCCTATTT
Ink4a TSS	5'-GTCCCTCCTTCCTTCCTCTG	5'-TCCTGAACCCTGCATCTCTT
Icos TSS	5'-ACACCACATCAACCTCCACA	5'-GGATGGCCACTCACCTGTTA
Cxcr5 upstream	5'-TCTCCCTTCCTTTCCTCCTC	5'-GCCCACTTCCTGTGTTGACT
Negative control	5'-GAGATGTAACTTTGCCATTTGC	5'-TCTTTTGCTCATTGCCCAG
