# Supplementary material for: Ezh2 programs TFH differentiation by integrating phosphorylation-dependent activation of Bcl6 and polycomb-dependent repression of p19Arf
Source: Nat Commun. 2018 Dec 21;9:5452. doi: 10.1038/s41467-018-07853-z (PMC6303346; doi:10.1038/s41467-018-07853-z)
Supplement: Supplementary file 3 — Reporting Summary [file 41467_2018_7853_MOESM3_ESM.pdf]

nature research

Corresponding author(s): Hai-Hui Xue

Reporting Summary

Nature Research wishes to improve the reproducibility of the work that we publish. This form provides structure for consistency and transparency in reporting. For further information on Nature Research policies, see Authors & Referees and the Editorial Policy Checklist.

Statistical parameters

When statistical analyses are reported, confirm that the following items are present in the relevant location (e.g. figure legend, table legend, main text, or Methods section).

n/a	Confirmed	
☐	☒	The exact sample size (*n*) for each experimental group/condition, given as a discrete number and unit of measurement
☐	☒	An indication of whether measurements were taken from distinct samples or whether the same sample was measured repeatedly
☐	☒	The statistical test(s) used AND whether they are one- or two-sided<br>*Only common tests should be described solely by name; describe more complex techniques in the Methods section.*
☐	☒	A description of all covariates tested
☐	☒	A description of any assumptions or corrections, such as tests of normality and adjustment for multiple comparisons
☐	☒	A full description of the statistics including central tendency (e.g. means) or other basic estimates (e.g. regression coefficient) AND variation (e.g. standard deviation) or associated estimates of uncertainty (e.g. confidence intervals)
☐	☒	For null hypothesis testing, the test statistic (e.g. *F*, *t*, *r*) with confidence intervals, effect sizes, degrees of freedom and *P* value noted<br>*Give P values as exact values whenever suitable.*
☒	☐	For Bayesian analysis, information on the choice of priors and Markov chain Monte Carlo settings
☒	☐	For hierarchical and complex designs, identification of the appropriate level for tests and full reporting of outcomes
☒	☐	Estimates of effect sizes (e.g. Cohen's *d*, Pearson's *r*), indicating how they were calculated
☐	☒	Clearly defined error bars<br>*State explicitly what error bars represent (e.g. SD, SE, CI)*

Our web collection on statistics for biologists may be useful.

Software and code

Policy information about availability of computer code

Data collection	No software was used for data collection
Data analysis	MACS2, SICER, FastQC, Tophat, Cuffdiff, Bowtie2, GSEA, Flowjo, and UCSC genome browser were used for data analysis and visualization

For manuscripts utilizing custom algorithms or software that are central to the research but not yet described in published literature, software must be made available to editors/reviewers upon request. We strongly encourage code deposition in a community repository (e.g. GitHub). See the Nature Research guidelines for submitting code & software for further information.

Data

Policy information about availability of data

All manuscripts must include a data availability statement. This statement should provide the following information, where applicable:

- Accession codes, unique identifiers, or web links for publicly available datasets
- A list of figures that have associated raw data
- A description of any restrictions on data availability

Data availability. The RNA-Seq data on WT and Ezh2–/– TFH cells, along with Tcf1, Ezh2, H3K27ac and H3K27me3 ChIP-Seq in WT TFH cells are deposited at the Gene Expression Omnibus under accession number GSE103387.

https://www.ncbi.nlm.nih.gov/geo/query/acc.cgi?acc=GSE103387

Field-specific reporting

Please select the best fit for your research. If you are not sure, read the appropriate sections before making your selection.

☒ Life sciences ☐ Behavioural & social sciences ☐ Ecological, evolutionary & environmental sciences

For a reference copy of the document with all sections, see nature.com/authors/policies/ReportingSummary-flat.pdf

Life sciences study design

All studies must disclose on these points even when the disclosure is negative.

Sample size	Sample size was chosen according to standard practices in the field. RNA-Seq samples were prepared in two replicates. ChIP-seq exoeriments were perform in one replicate, following by ChIP-qPCR validation of binding events of interest. All biochemical analyses were performed 2 to 3 independent experiments as described in figure legends. No statistical methods were used to predetermine sample sizes.
Data exclusions	No data were excluded.
Replication	All biochemical and phenotypic analyses were performed in at least two replicates as described in figure legends. Data from multiple experiments and multiple replicates were analyzed for statistical significance.
Randomization	Samples were grouped by genotype. No randomization was performed.
Blinding	No blinding was performed in this st

Reporting for specific materials, systems and methods

Materials & experimental systems

n/a	Involved in the study	
☒	☐	Unique biological materials
☐	☒	Antibodies
☐	☒	Eukaryotic cell lines
☒	☐	Palaeontology
☐	☒	Animals and other organisms
☒	☐	Human research participants

Methods

n/a	Involved in the study	
☐	☒	ChIP-seq
☐	☒	Flow cytometry
☒	☐	MRI-based neuroimaging

Antibodies

Antibodies used	anti-CD4 (RM4-5), anti-CD44 (IM7), anti-CD62L (MEL-14), anti-CD69 (H1.2F3), anti-CD45.2 (104), anti-ICOS (C398.4A), anti-CD25 (PC61.5), Thy1.1 (HIS51), anti-PD-1 (J43), anti-Fas (15A7), anti-GL7 (GL7), anti-IgD (11-26), anti-CD138 (281-2), anti-T-bet (eBio4B10), and rat IgG2a κ isotype control (eBR2a, for intracellular staining of Bcl6) were from eBiosciences; anti-Bcl6 (K112-91) and anti-Ezh2 (11/Ezh2) from BD Biosciences; anti-Irf4 (IRF4.3E4) from BioLegend; anti-Tcf1 (C63D9) and isotype control (Cat. No. 4410S for intracellular staining of Ezh2 and Tcf1) from Cell Signaling Technology; and anti-SLAM (TC15-12F12.2) from BioLegend. pS21-Ezh2 (rabbit polyclonal, Bethyl Laboratories) or pT487-Ezh2 (rabbit polyclonal, abbexa, UK), anti-FLAG (clone M2, Sigma-Aldrich), anti-HA (F7, Santa Cruz Biotechnology), anti-Bcl6 (D65C10, Cell Signaling Technologies), anti-p19Arf (5-C3-1, Novus Biologicals), anti-Tcf1 (C63D9, Cell Signaling Technologies), anti-H3K27me3 (Millipore, 17-622) or anti-H3K27ac (Abcam, ab4729), anti-Ezh2 (Cat. No. ab3748, Abcam).
Validation	Antibodies were validated by manufacturer, and also in others' studies.

Eukaryotic cell lines

Policy information about cell lines

Cell line source(s)	ATCC
Authentication	validated by the provider.

Mycoplasma contamination	confirmed to be negative for mycoplasma.
Commonly misidentified lines<br>(See ICLAC register)	not applicable.

Animals and other organisms

Policy information about studies involving animals; ARRIVE guidelines recommended for reporting animal research

Laboratory animals	All mice analyzed were 6–12 weeks of age, and both genders were used. All mouse experiments were performed under protocols approved by the Institutional Animal Use and Care Committees of the University of Iowa.
Wild animals	No wild animals were used in this study.
Field-collected samples	No field-collected samples were used int his study.

ChIP-seq

Data deposition

☒ Confirm that both raw and final processed data have been deposited in a public database such as GEO.

☒ Confirm that you have deposited or provided access to graph files (e.g. BED files) for the called peaks.

Data access links<br>*May remain private before publication.*	https://www.ncbi.nlm.nih.gov/geo/query/acc.cgi?acc=GSE103387
Files in database submission	GSM2769711 Day8 WT Tfh, Ezh2 ChIP-Seq<br>GSM2769712 Ezh2-deficient naïve CD4+, Ezh2 ChIP-Seq<br>GSM2769713 Day8 WT Tfh, Tcf1 ChIP-Seq<br>GSM2769714 Tcf1-deficient CD4+, Tcf1 ChIP-Seq<br>GSM2769715 Day8 WT Tfh rep1<br>GSM2769716 Day8 WT Tfh rep2<br>GSM2769717 Day8 Ezh2-deficient Tfh rep1<br>GSM2769718 Day8 Ezh2-deficient Tfh rep2
Genome browser session<br>(e.g. UCSC)	not applicable

Methodology

Replicates	Two biological replicates were used for RNA-Seq, and one replicate was analyzed for ChIP-Seq.
Sequencing depth	For ChIP-seq and RNA-seq datasets, 30-50 million raw reads were sequenced. Samples were sequenced as 50bp single end reads.
Antibodies	anti-H3K27me3 (Millipore, 17-622) or anti-H3K27ac (Abcam, ab4729), anti-Ezh2 (Cat. No. ab3748, Abcam). Tcf1 antibody was generated in-house.
Peak calling parameters	Mapped reads were processed with SICER (v1.1) for peak calling with the setting of FDR < 10–4.
Data quality	The sequencing quality of ChIP-Seq libraries was assessed by FastQC.
Software	FastQC, Bowtie2 v2.2.6, and SICER.

Flow Cytometry

Plots

Confirm that:

☒ The axis labels state the marker and fluorochrome used (e.g. CD4-FITC).

☒ The axis scales are clearly visible. Include numbers along axes only for bottom left plot of group (a 'group' is an analysis of identical markers).

☒ All plots are contour plots with outliers or pseudocolor plots.

☒ A numerical value for number of cells or percentage (with statistics) is provided.

Methodology

Sample preparation	Lymph nodes or spleens were dissociated into FACS buffer (2%FBS), passed through 40uM filters, and stained for indicated markers.

Instrument	BD FACSAria Fusion, BD FACSAriaII for cell sorting, and BD FACSVerse and LSR II for data acquisition.
Software	Flowjo was used for data analysis and BD FACSVerse was used for data acquisition.
Cell population abundance	Sorted populations were at approximately 95% on post-sorting analysis.
Gating strategy	Starting cells were gated by FSC/SSC gates, then on SSC-width to exclude doublets.

☒ Tick this box to confirm that a figure exemplifying the gating strategy is provided in the Supplementary Information.
