# Supplementary material for: Ezh2 programs TFH differentiation by integrating phosphorylation-dependent activation of Bcl6 and polycomb-dependent repression of p19Arf
Source: Nat Commun. 2018 Dec 21;9:5452. doi: 10.1038/s41467-018-07853-z (PMC6303346; doi:10.1038/s41467-018-07853-z)
Supplement: Supplementary file 2 — Peer Review File [file 41467_2018_7853_MOESM2_ESM.pdf]

Editorial Note: This manuscript has been previously reviewed at another journal that is not operating a transparent peer review scheme. This document only contains reviewer comments and rebuttal letters for versions considered at Nature Communications.

REVIEWERS' COMMENTS:

Reviewer #1 (Remarks to the Author):

I can see that the revised version has dealt with the points raised by reviewer #2 with dedicated efforts. This work warrants publication in Nature communication.

**Response to Reviews:**

Reviewer #1 (Remarks to the Author):

**I can see that the revised version has dealt with the points raised by reviewer #2 with dedicated efforts. This work warrants publication in Nature communication.**

We thank the Reviewer for commending our revision effort and the support for publication.
